# Combined immunization with SARS-CoV-2 spike and SARS-CoV nucleocapsid protects K18-hACE2 mice but increases lung pathology

**DOI:** 10.1038/s41541-025-01085-1

**Published:** 2025-02-13

**Authors:** Jaekwan Kim, Alla Kachko, Prabhuanand Selvaraj, David Rotstein, Charles Brandon Stauft, Naveen Rajasagi, Yangqing Zhao, Tony Wang, Marian Major

**Affiliations:** 1 Division of Viral Products, Center for Biologics Evaluation and Research, Food and Drug Administration, Silver Spring, MD USA; 2 Division of Food Compliance, Center for Veterinary Medicine, Food and Drug Administration, Rockville, MD USA

**Keywords:** Vaccines, Infectious diseases

## Abstract

Vaccines against SARS-CoV-2 have targeted the spike protein and have been successful at preventing disease. However, with the emergence of variants, spike-specific vaccines become less effective. The nucleocapsid protein is relatively conserved among variants of SARS-CoV-2 and is a candidate for addition to spike in next generation vaccines for the induction of T cell protection. Previous studies on SARS-CoV have suggested that the induction of an immune response to nucleocapsid could result in enhanced disease. Using the K18-hACE2 mouse model we investigated immunization with a variant nucleocapsid, from SARS CoV (N1) alone or in combination with spike from SARS-CoV-2 and compared this to nucleocapsid from SARS-CoV-2 (N2). The spike-nucleocapsid-based vaccines conferred protection against SARS-CoV-2 in lungs and brain and decreased lung pathology compared to control mice. However, higher T and B cell immune responses were observed in N1-immunized mice prior to challenge, whether delivered alone or with spike, and immunization with N1 resulted in increased lung pathology compared to immunization with spike or N2. These findings suggest that spike-nucleocapsid-based vaccines are safe and effective, even with variant nucleocapsid sequences, but that viral control in this mouse model may be associated with higher lung pathology, compared to spike immunization alone, due to the immunogenic qualities of the nucleocapsid antigen.

## Introduction

Severe acute respiratory syndrome coronavirus 2 (SARS-CoV-2) has infected more than 770 million people, with over 7 million deaths worldwide as of December 2024^1,2^. Vaccines targeting the spike (S) protein have been highly successful at preventing hospitalization and severe disease^3–5^. However, the S protein mutates over time, with variants of concern emerging at intervals with the potential to evade the immune responses induced by current vaccines^6–8^. The induction of broader and more enduring immune responses by vaccination is critical to preventing further outbreaks. Coronaviruses (CoV) encode four main structural proteins, S, membrane, envelope and nucleocapsid (N)^9^. The primary function of the N protein is to package the viral RNA genome and interact with the membrane proteins for virion assembly^10^. The N protein is highly immunogenic^11,12^, more conserved than S and represents a useful candidate as an additional target for inclusion in future next-generation vaccines^13^. The N protein is not presented on the virion surface, although it has been shown to be expressed on the surface of infected cells^14^, and the main mechanism of N-mediated protective immunity would be through the engagement of innate immune cells or targeting of infected cells through the induction of T cells. CD8+ cytotoxic T cells are pivotal in recognizing viral peptides presented on infected cells and executing their elimination. The orchestration of this response involves the release of cytokines, such as interferon-gamma (IFN-γ), tumor necrosis factor-alpha (TNF-α), and interleukins, which collectively amplify the immune reaction and contribute to antiviral defenses. T cells have been shown to be induced during SARS-CoV-2 infection and vaccination with S^15–19^. A recent publication demonstrated antibody-independent protection against SARS-CoV-2 from T cell immunity to the S antigen in mouse models^20^, further emphasizing the role that T cells can play in protection from SARS-CoV-2 infections. Several studies have been performed testing N either alone or in combination with other proteins, including S, in preclinical models for COVID-19 vaccines^21–25^. Recently it was shown that N combined with S for immunization of mice had a synergistic effect on protection from virus challenge and dissemination of the virus to the brain^24^. However, the use of the N antigen in vaccines presents concerns as previous studies on SARS-CoV suggested that the induction of an immune response to N could result in enhanced disease following infection with the virus^26^. In addition, despite the relative sequence conservation of the N protein among CoV isolates, adaptive mutations have been shown to arise^27,28^. The advantage of adding a T cell-based vaccination strategy is that T cell epitope escape in one individual will not necessarily result in T cell escape in another individual with a different MHC haplotype. However, a mismatch of the N protein used for vaccination with the circulating virus could lead to a sub-optimal response which could result in enhanced disease following exposure to SARS-CoV-2. In this study, we have explored the use of a mismatched N protein in protection and disease enhancement in the K-18-hACE2 transgenic mouse model using adenovirus vectors expressing the N protein of SARS CoV (Ad-N1) and of SARS CoV-2 (Ad-N2) delivered either alone or in combination with adenovirus expressing the SARS CoV-2 S antigen. We chose to use the N protein from the 2004 SARS-CoV instead of a sequence from a SARS-CoV-2 variant in part due to the previous reports of enhanced disease in mouse models with this antigen^26^, but also to study a more diverse antigenic sequence. Thus far, SARS-CoV-2 variants have exhibited only low-level diversity in the N protein (0-1.9%) (Supplementary Table 1). The use of the SARS-CoV N sequence with a 9.8% difference from the SARS-CoV-2 N protein (Supplementary Table 1) represents a worst-case scenario for immunization with a nucleocapsid-containing vaccine that does not match the infecting virus. In the absence of neutralizing antibody, we observed lower mortality and reduced lung pathology in Ad-N1 and Ad-N2 immunized mice following intranasal challenge with SARS-CoV-2, combined with better virus control, compared to control mice. The addition of Ad-S for immunization improved protection with reduced lung pathology, lower virus titers in lungs and brains of challenged mice and 100% survival. However, we observed increased lung pathology in mice immunized with Ad-N1 or Ad-N1 plus Ad-S compared to those immunized with Ad-N2 plus Ad-S or Ad-S alone. Overall, our data indicate that the inclusion of N in a vaccine can result in protection from SARS-CoV-2 infection even when the N antigen sequence is not homologous with the infecting virus, but this protection may be associated with higher inflammatory cell infiltration in the lungs.

## Results

### Ad-N1 and Ad-N2 induce SARS-CoV-2 specific immune responses in K18-hACE2 mice

**Fig. 1:**
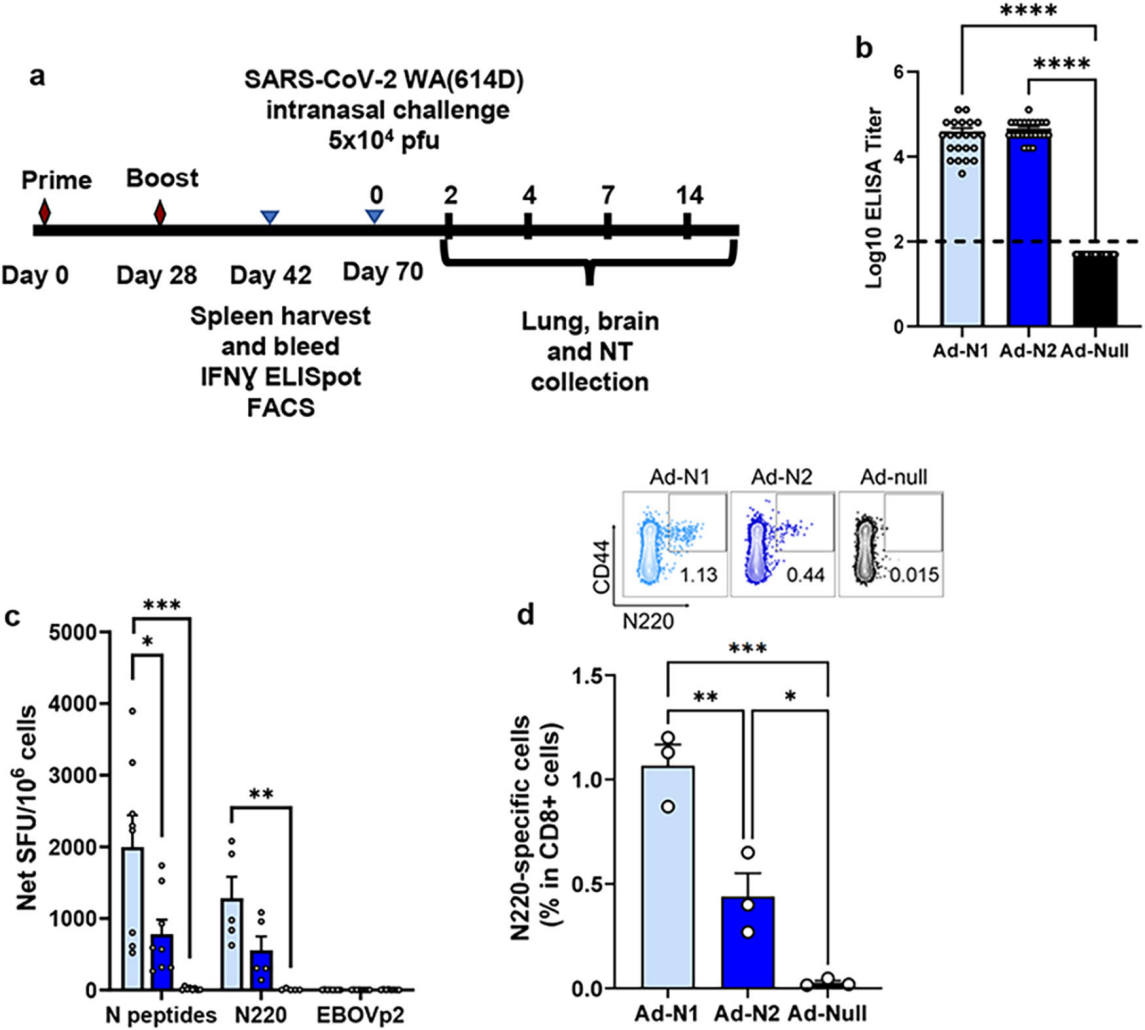
Immunization scheme and immunogenicity of Ad-N1 and Ad-N2. **a** Schedule of vaccination, virus challenge, and harvesting of analytes. **b** Nucleocapsid binding antibody endpoint titers in K18-hACE2 mice 2 weeks post boost. Titers falling below the lowest dilution of 1:100, as indicated by the dashed line, were assigned a value of 50. **c** Frequencies of IFN-γ expressing splenocytes after stimulation with overlapping peptides representing the N protein, N220 peptide, or a pool of Ebola virus peptides (EBOVp2) were assessed using ELIspot. **d** Frequencies of SARS-CoV-2 N220-specific CD8 T cells in freshly isolated splenocytes assessed using a dextramer. Error bars represent SEM. Each dot represents an individual mouse. Statistical analysis was conducted using one-way ANOVA with Tukey’s Multiple Comparisons. Significance levels are indicated as follows: *p < 0.05, **p < 0.01, ***p < 0.001, ****p < 0.0001. For clarity, comparisons that were not significant are not shown.

Immunogenicity of adenovirus constructs expressing the N protein of SARS-CoV (Ad-N1) or SARS-CoV-2 (Ad-N2) were tested in a prime-boost regimen with intramuscular inoculations on days 0 and 28 (Fig. 1a). Sera and spleens were collected 2 weeks after the second dose and analyzed for antibodies and T cell responses. Mice immunized with either Ad-N1 or Ad-N2 developed SARS-CoV-2 N binding antibodies (>4 log10 end point titer) (Fig. 1b), while no signal above background was observed in mice immunized with an Ad5 vector lacking a transgene (Ad-Null).

An IFN-γ ELIspot assay using overlapping peptide pools spanning the SARS-CoV-2 N sequence and a single N220 peptide previously shown to be a CD8+ T cell epitope in C57BL/6 mice^29^, demonstrated the induction of T cell responses in the Ad-N1 and Ad-N2 immunized mice (Fig. 1c). The dominant T cell response was to the peptide pool containing the N220 peptide and is consistent with the conservation of this epitope sequence in both the Ad-N1 (from SARS-CoV) and Ad-N2 (from SARS-CoV-2) constructs (Supplementary Fig. 1). Interestingly, we observed significantly higher IFN-γ ELIspot responses in the Ad-N1 immunized mice compared with the Ad-N2 immunized mice using peptide pools. Responses were also higher against the N220 peptide, but these did not reach significance compared to the Ad-N2 immunized mice. N220-specific CD8+ T-cells were detected in both groups using a N220 dextramer (Fig. 1d), the levels of dextramer-positive CD8+ T cells in the Ad-N1 group were significantly higher than those observed for the Ad-N2 group.

### Higher CD8+ T cell responses are induced by Ad-N1 and higher CD4+ T cell responses are induced by Ad-N2

**Fig. 2:**
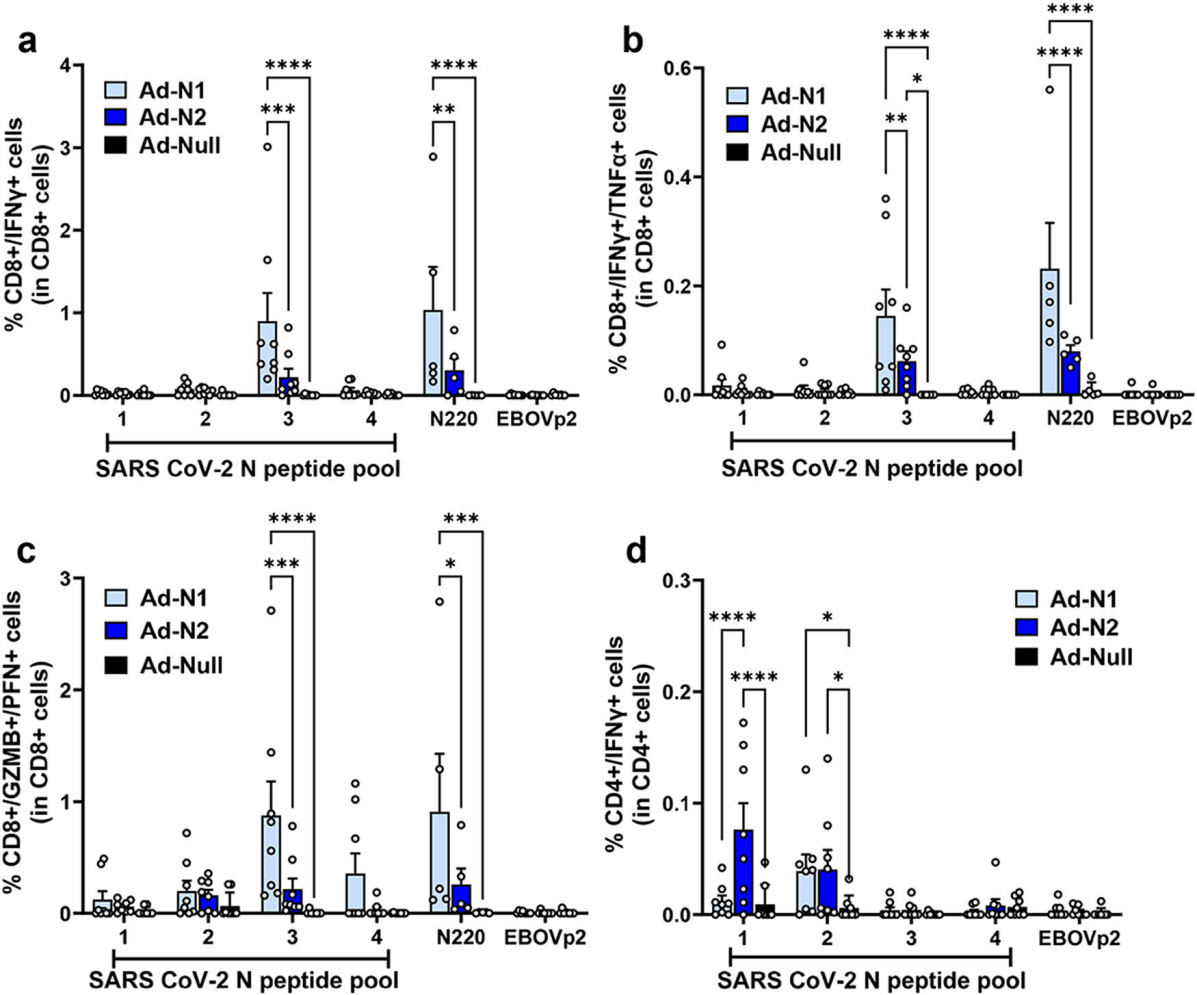
Flow cytometry analysis of splenocytes from Ad-N1, Ad-N2, Ad-Null immunized mice. Spleens were collected at 2 weeks post boost and analyzed by flow cytometry following in vitro stimulation with overlapping peptides of the SARS-CoV-2 N or a pool of Ebola virus antigen (EBOVp2). **a** Frequencies of CD8+ cells expressing IFN-γ following SARS CoV-2 N peptide stimulation. **b** Frequencies of CD8+ cells expressing both IFN-γ and TNF-α following SARS CoV-2 N peptide stimulation **c** Frequencies of CD8+ cells expressing both Granzyme B and Perforin following SARS CoV-2 N peptide stimulation. **d** Frequencies of CD4+ cells expressing IFN-γ SARS CoV-2 N peptide stimulation. Representative flow cytometry dot plots from one mouse per group are shown in Supplementary Fig. 2. Error bars represent SEM. Each dot represents an individual mouse. Statistical analysis was conducted using two-way ANOVA with Tukey’s Multiple Comparisons. Significance levels are indicated as follows: *p < 0.05, **p < 0.01. For clarity, comparisons that were not significant are not shown.

Using flow cytometry analysis N-specific CD4+ and CD8+ T cell responses were detected in the Ad-N1 and Ad-N2 immunized mice (Fig. 2a–d and Supplementary Fig. 2). CD8 + IFN-γ-producing cells were detected in both immunized groups using the same SARS-CoV-2 peptide pools used for the ELIspot analysis (Fig. 2a). As observed in the ELIspot studies, a significantly higher frequency of IFN-γ-positive CD8+ cells was detected in the Ad-N1 group compared with the Ad-N2 group following stimulation with peptide pools and a higher level was observed following stimulation with the N220 peptide alone (Fig. 2a). CD8+ T cells producing both IFN-γ and TNF-α (Fig. 2b) and granzyme-B and perforin (Fig. 2c) were also induced in both immunized groups of mice, with significantly higher levels observed in the Ad-N1 immunized group.

We were able to detect CD4+ T cells by flow cytometry in both immunized groups of mice, though at a lower frequency than seen for CD8+ cells (Fig. 2d). In the case of CD4 + IFN-γ-producing cells, we observed a significantly higher frequency in the Ad-N2 immunized mice, compared with the Ad-N1 group (Fig. 2d), while low levels of CD4+ cells producing IL4 were detected in all groups (Supplementary Fig. 3), indicating the induction of a Th1-type response in immunized mice.

### Ad-N1 and Ad-N2 provide low-level protection in SARS-CoV-2 challenged mice

**Fig. 3:**
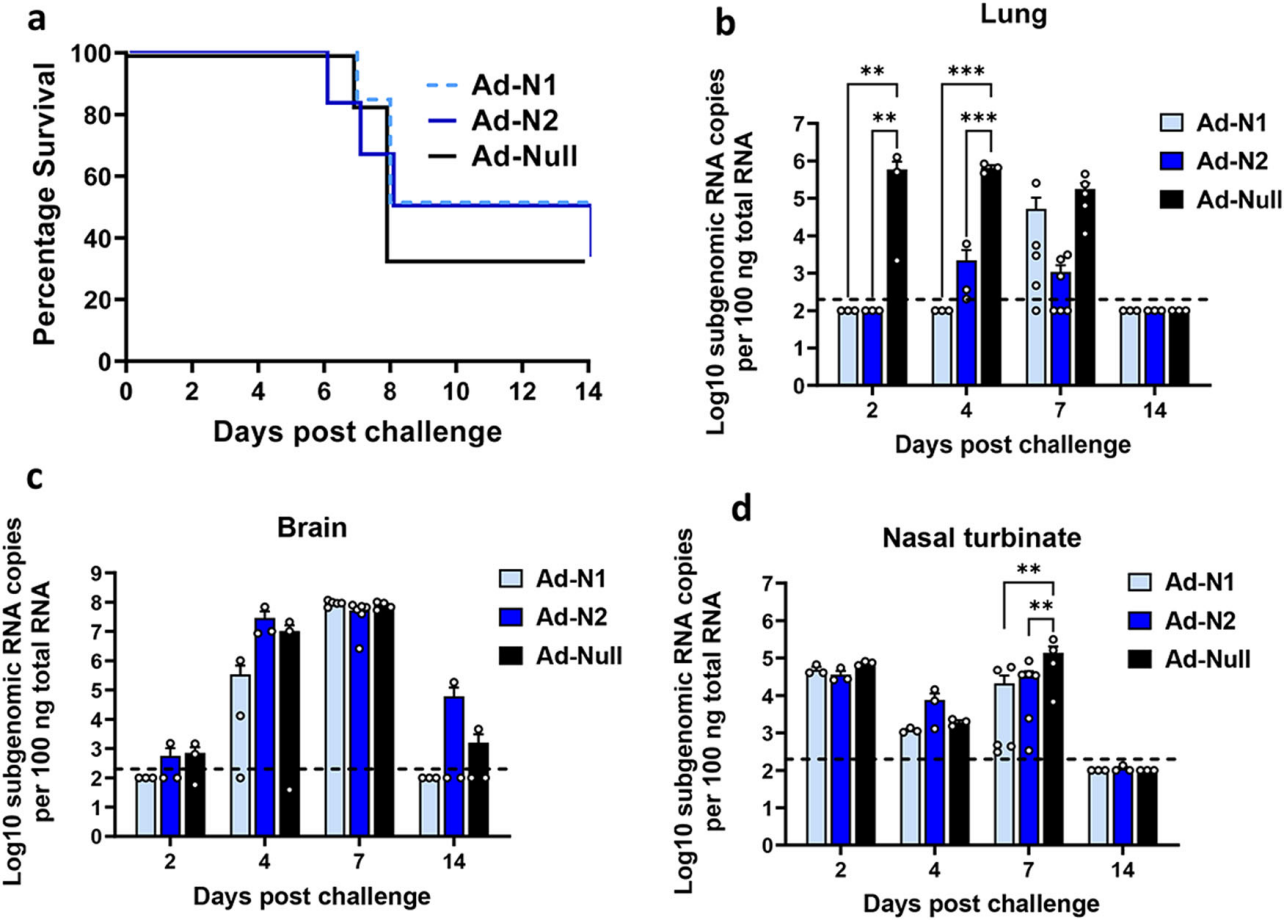
Survival and sgRNA levels in Ad-N1 and Ad-N2 immunized mice post SARS-CoV-2 challenge. **a** Kaplan–Meier survival curve of SARS-CoV-2-challenged K18-hACE2 mice. **b**-**d** Subgenomic viral RNA (sgRNA) levels in the lungs (**b**), brains (**c**), and nasal turbinate (**d**) of challenged mice. sgRNA copy numbers falling below the limit of detection (LOD) (200 copies), indicated by the dashed line, were set as half of the LOD. Error bars represent SEM, each dot represents an individual mouse. Statistical analysis was conducted using two-way ANOVA with Tukey’s Multiple Comparisons. Significance levels are indicated as follows: **p < 0.01, ***p < 0.001. For clarity, comparisons that were not significant are not shown.

To assess the protective effect of immune responses induced against Ad-N1 or Ad-N2, K18-hACE2 mice were challenged 6 weeks after the 2^nd^ dose of vaccine with 5×10^4^ PFU SARS-CoV-2 (Washington strain, WA1/2020 (614D)) (Fig. 1a). This virus expresses N antigen homologous to the N protein encoded by the Ad-N2 recombinant. Ad-N1 and Ad-N2 immunized mice showed slightly better survival compared to Ad-Null immunized mice (Fig. 3a), although this was not found to be significant. Viral subgenomic RNA (sgRNA) titers in the lung, brain and nasal turbinate were assessed on days 2, 4, 7 and 14 post challenge. We observed significantly lower levels of sgRNA in the lungs of both Ad-N1 and Ad-N2 immunized mice at days 2 and 4 post challenge (Fig. 3b) compared with Ad-Null immunized mice. We also observed a lower level of sgRNA in Ad-N2 immunized mice at day 7 post-challenge. By day 14 post-challenge there was no detectable sgRNA in the lungs of any challenged mice (Fig. 3b). In contrast, we observed no significant differences in the levels of sgRNA in the brains (Fig. 3c) and only at day 7 in the nasal turbinates (Fig. 3d) for the immunized groups post-challenge compared to the Ad-Null group.

**Fig. 4:**
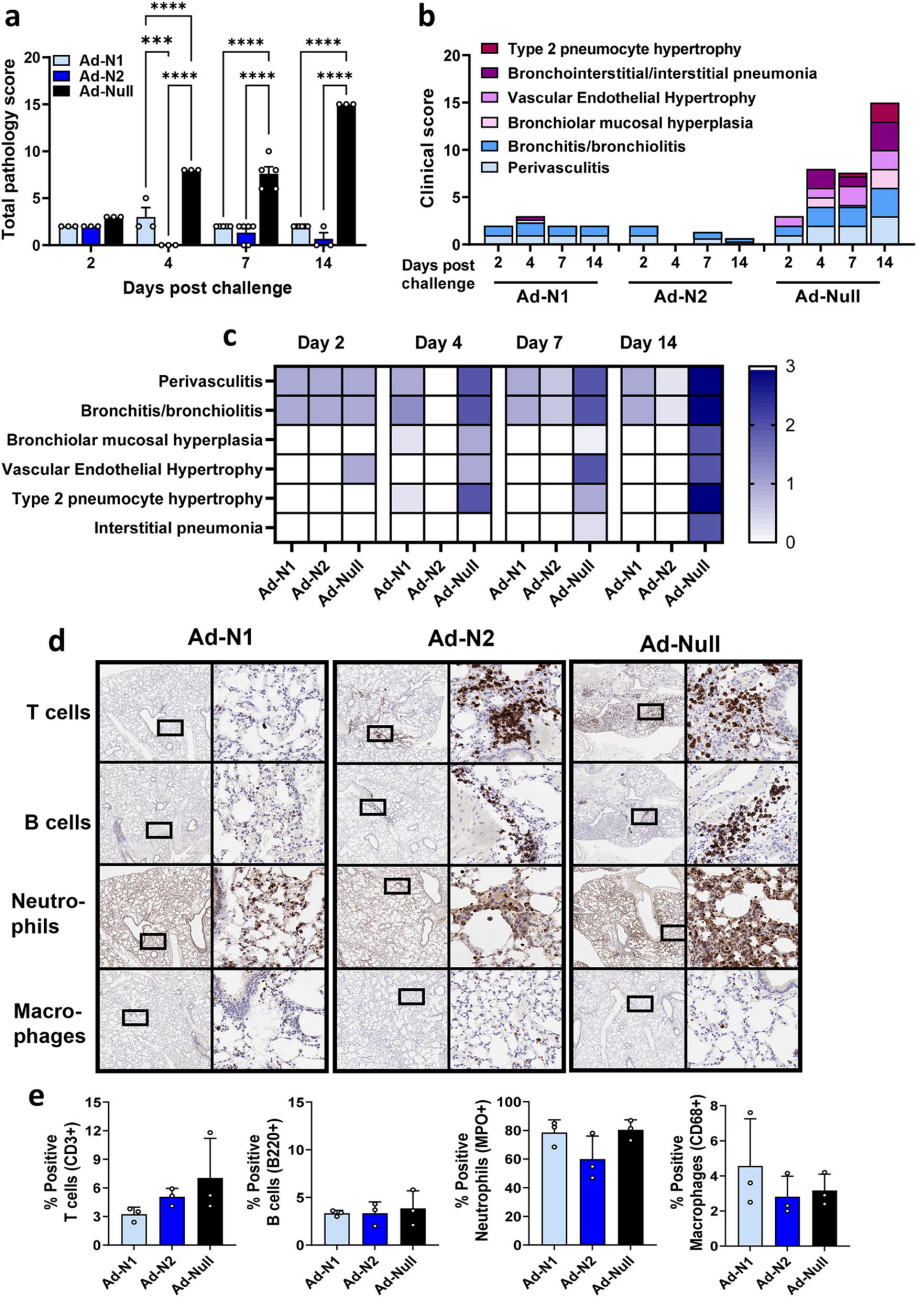
Histopathology analysis of Ad-N1 and Ad-N2 immunized mice following SARS-CoV-2 challenge. Hematoxylin and eosin (H&E) staining was performed on paraffin-embedded lung tissues of mice in each group on specified days post-challenge. **a** Mean total pathology score for individual groups. The combined scores of six parameters for lung pathology are presented. **b** Mean inflammatory scores for each of six lung pathology parameters for individual groups. **c** Heatmap showing inflammatory levels in immunized mice post-challenge. **d** Immunohistochemistry staining for T cells, B cells, neutrophils and macrophages in lungs of immunized mice at 7 days post-challenge. Images are shown at low (2x) and high (20x) magnification. The box in the low-magnification image represents the area shown in the high-magnification image. **e** Percentage of positive T cells, B cells, neutrophils and macrophages in total scanned lung tissues. Error bars represent SEM, each dot represents an individual mouse. Statistical analysis was conducted using two-way ANOVA (**a**) or one-way ANOVA (**e**) with Tukey’s Multiple Comparisons. Significance levels are indicated as follows: ***p < 0.001, ****p < 0.0001. For clarity, comparisons that were not significant are not shown.

Inflammation and pathology scores were assessed in hematoxylin and eosin (H&E)-stained lung tissue sections obtained from challenged mice at days 2, 4, 7, and 14 (Fig. 4a). Significantly lower levels of inflammatory infiltration were observed in the Ad-N1 and Ad-N2 immunized groups throughout the follow-up period compared to the Ad-Null group (Fig. 4a). However, we observed elevated inflammatory infiltration in the Ad-N1 immunized mice compared to the Ad-N2 immunized mice at days 2 through 14 (Fig. 4a–c). Mild perivasculitis and bronchitis were observed in the Ad-N1 and Ad-N2 immunized groups, while Ad-Null mice exhibited intermediate symptoms (Fig. 4b, c and Supplementary Fig. 4). Starting from day 2, Ad-Null mice exhibited increased cellularity (hyperplasia/hypertrophy), whereas the Ad-N1 and Ad-N2 groups showed minimal symptoms. The progression of lung damage, including type 2 pneumocyte hypertrophy and interstitial pneumonia, was observed to increase in the Ad-Null group while these manifestations were barely detectable in the Ad-N1 and Ad-N2 groups (Fig. 4b, c and Supplementary Fig. 4). We performed immunohistochemistry staining of lung tissues from day 7 post-challenge for T cells, B cells, neutrophils and macrophages. We observed increased T and B cell infiltration in the lungs of Ad-Null mice compared to the N-immunized groups (Fig. 4d) and increased clusters of T and B cells in Ad-N2 immunized mice compared to Ad-N1 immunized mice (Fig. 4d). Although quantification of cell numbers in tissue sections did not show significant increases in total numbers of T or B cells in any group (Fig. 4e), we observed 1.5-2-fold higher levels of T cells in the Ad-N2 and Ad-Null groups compared with the Ad-N1 group. We observed high levels of neutrophil infiltrates in all three groups of challenged mice (Fig. 4d, e) and similar levels of macrophages (Fig. 4d, e).

### Addition of N impacts S immune responses in combined immunization

Our observations using Ad-N1 to immunize mice challenged with SARS-CoV-2 suggested that immune responses to heterologous N antigen still results in low level protection with reduced pathology compared to control challenged mice. We next assessed immune responses, challenge outcome and lung pathology following immunization with Ad-N1 or Ad-N2 in combination with an adenovirus construct expressing S (Ad-S). Results were compared with mice immunized with Ad-S alone or Ad-Null.

**Fig. 5:**
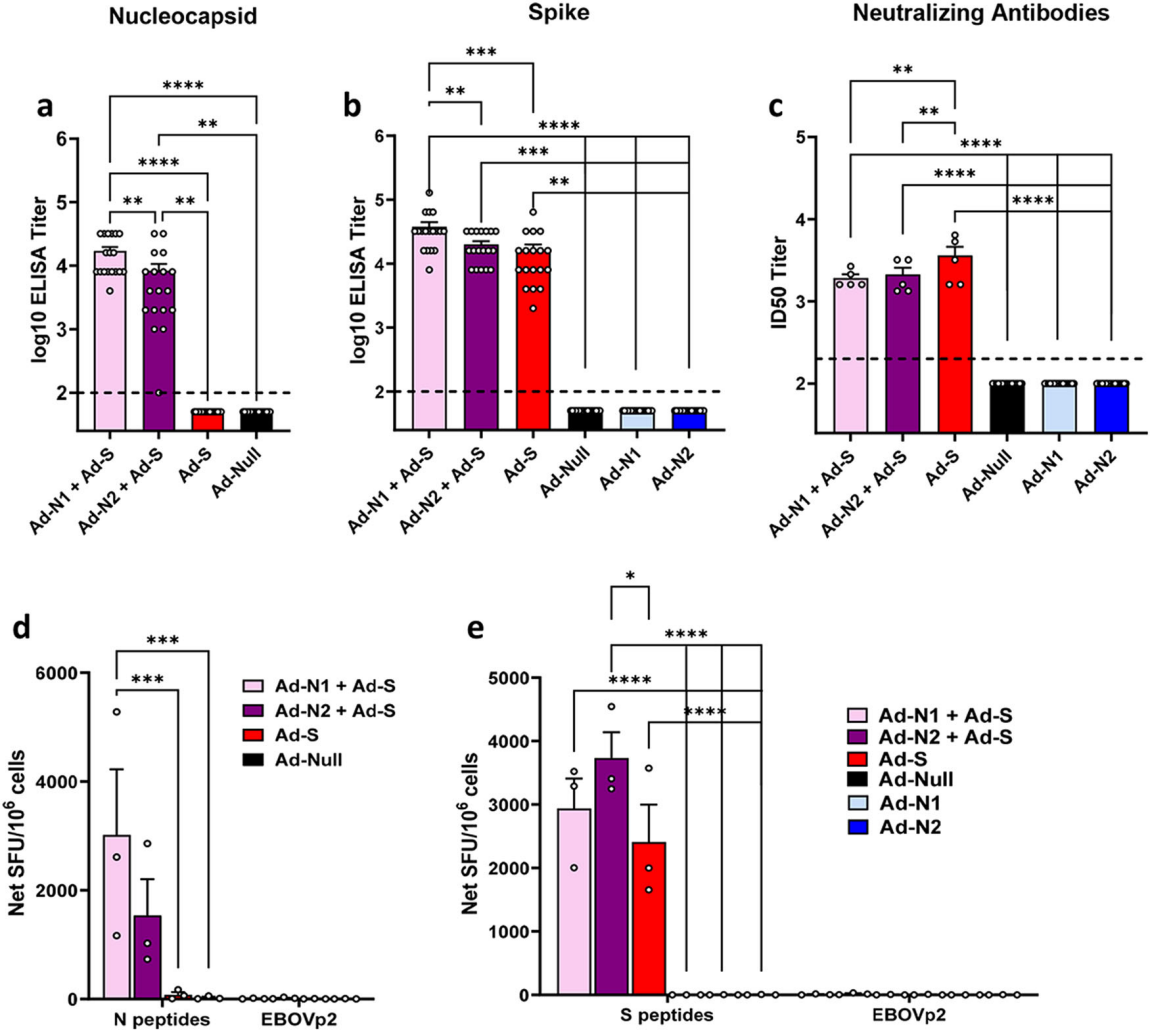
Immune responses against SARS-CoV-2 S and N antigens after immunization with Ad-N1 or Ad-N2 combined with Ad-S or Ad-S alone. N antigen (**a**) and S antigen (**b**) binding antibody endpoint titers in K18-hACE2 mice two weeks post-boost. Titers falling below the lowest dilution of 1:100, as indicated by the dashed line, were assigned a value of 50. **c** Neutralization titers of sera collected two weeks post-boost using SARSpp-WA1 pseudotyped virus. A titer of 100 was assigned to samples that did not show 50% inhibition at the lowest dilution of 1:200. **d** Frequencies of IFN-γ expressing splenocytes after stimulation with overlapping peptides representing the SARS-CoV-2 N protein or EBOVp2 peptide pool were assessed using ELISpot assay. **e** Frequencies of IFN-γ expressing splenocytes after stimulation with overlapping peptides representing the SARS-CoV-2 S protein or EBOVp2 peptide pool were assessed using ELISpot assay. Error bars represent SEM, and each dot represents an individual mouse. Statistical analysis was conducted using one-way ANOVA with Tukey’s Multiple Comparisons. Significance levels are indicated as follows: **p < 0.01, ***p < 0.001, ****p < 0.0001. For clarity, comparisons that were not significant are not shown.

Binding assays showed that antibody responses specific to the test antigen were induced in all groups of immunized mice (Fig. 5a, b). No N-specific antibodies were detected in the Ad-S immunized group (Fig. 5a). Results for mice immunized with Ad-N1 and Ad-N2 alone are included to demonstrate that there is no cross-reactivity of N antibodies in combined immunizations (Fig. 5b and c). We observed significantly higher levels of S and N binding antibodies in the Ad-N1+Ad-S groups compared with the Ad-N2+Ad-S and Ad-S immunized mice (Fig. 5a, b) although we found significantly lower levels of neutralizing antibodies in the Ad-N+Ad-S immunized mice, whether with Ad-N1 or Ad-N2 compared with S alone (Fig. 5c).

ELIspot analysis of IFN-γ production in cells stimulated with SARS-CoV-2 N or SARS-CoV-2 S peptide pools also showed specific responses in all immunized groups (Fig. 5d, e). Similar to that seen for the Ad-N1 and Ad-N2 alone (Fig. 1), there was a higher frequency of IFN-γ producing cells in the Ad-N1 + Ad-S immunized mice following N peptide stimulation compared with the Ad-N2 + Ad-S immunized mice (Fig. 5d). Splenocytes from all mice immunized with Ad-S, whether alone or in combination with Ad-N, produced IFN-γ following stimulation with S peptide pools (Fig. 5e), with higher levels being observed in the combined immunization groups compared to Ad-S alone.

**Fig. 6:**
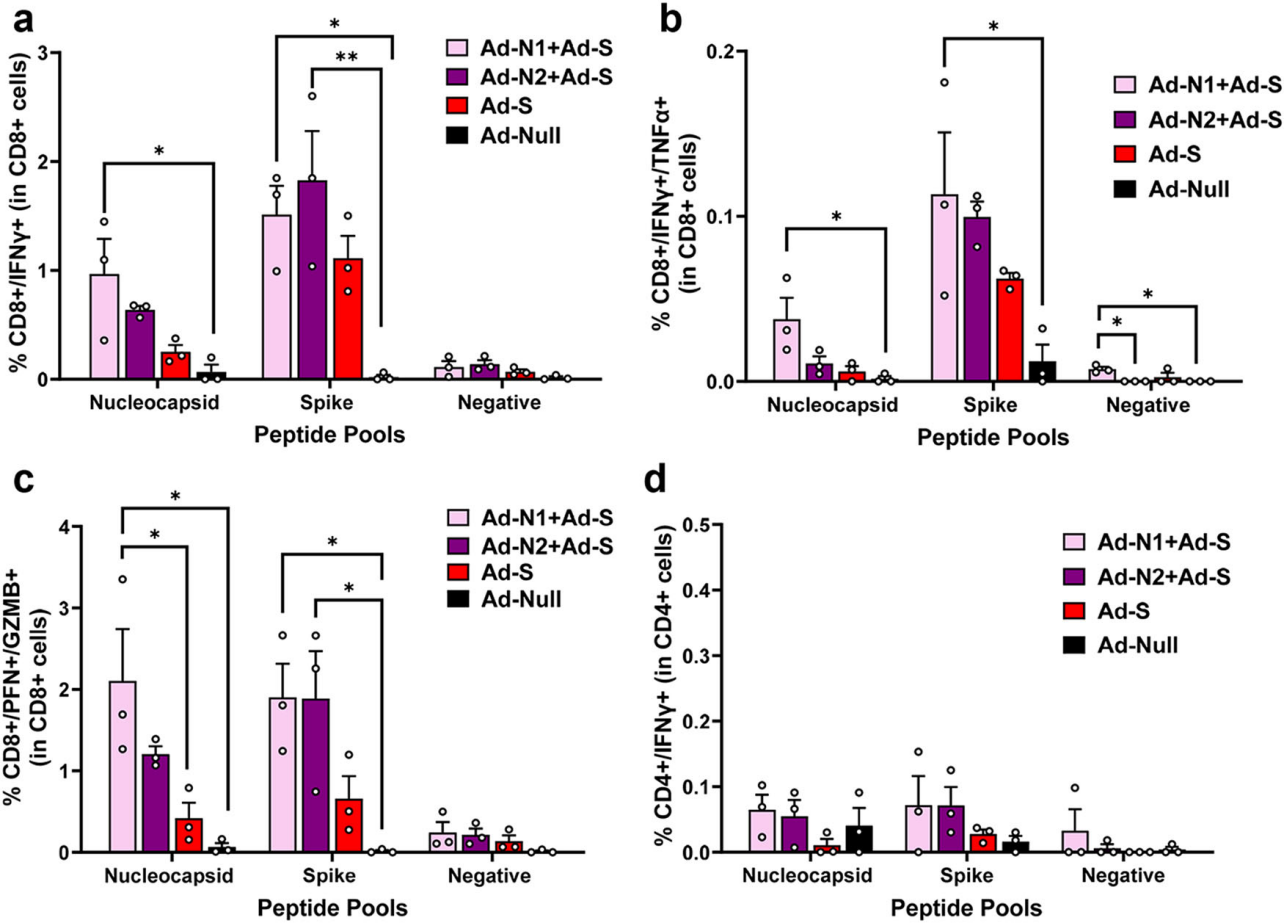
Flow cytometry analysis of splenocytes following combined immunization. Spleens were collected at 2 weeks post boost and analyzed by flow cytometry following in vitro stimulation with overlapping peptides of the SARS-CoV-2 N or S protein or a pool of Ebola virus antigen (EBOVp2). **a** Frequencies CD8+ cells expressing IFN-γ following SARS CoV-2 N and S peptide stimulation. **b** Frequencies CD8+ cells expressing both IFN-γ and TNF-α following SARS CoV-2 N and S peptide stimulation **c** Frequencies CD8+ cells expressing both Granzyme B and Perforin following SARS CoV-2 N and S peptide stimulation. **d** Frequencies CD4+ cells expressing IFN-γ SARS CoV-2 N and S peptide stimulation. Representative flow cytometry dot plots from one mouse per group are shown in Supplementary Fig. 5. Error bars represent SEM. Each dot represents an individual mouse. Statistical analysis was conducted using two-way ANOVA with Tukey’s Multiple Comparisons. Significance levels are indicated as follows: *p < 0.05, **p < 0.01. For clarity, comparisons that were not significant are not shown.

Using flow cytometry analysis N- and S-specific CD8+ T cell responses were detected in all immunized mice following peptide stimulation (Fig. 6 and Supplementary Fig. 5). As observed in the T cell studies using Ad-N1 and Ad-N2 alone, when cells were stimulated with N peptide pools a higher frequency of CD8+ cells producing IFN-γ (Fig. 6a), IFN-γ and TNF-α (Fig. 6b) and cells producing granzyme B and perforin (Fig. 6c) were observed in the mice immunized with Ad-N1+Ad-S compared with the mice immunized with Ad-N2+Ad-S, although the differences did not reach statistical significance. When splenocytes were stimulated with S peptide pools, CD8+ cells producing IFN-γ (Fig. 6a), IFN-γ and TNF-α (Fig. 6b) and granzyme B and perforin (Fig. 6c) were detectable in all Ad-S immunized groups. The levels in the Ad-N1+Ad-S and Ad-N2+Ad-S were consistently higher than the levels observed for the Ad-S only group. There was no specific detection of CD4+ cells producing IFN-γ for any of the immunized groups (Fig. 6d).

### Combined Ad-N and Ad-S immunization results in survival but increased lung pathology compared to Ad-S immunization

**Fig. 7:**
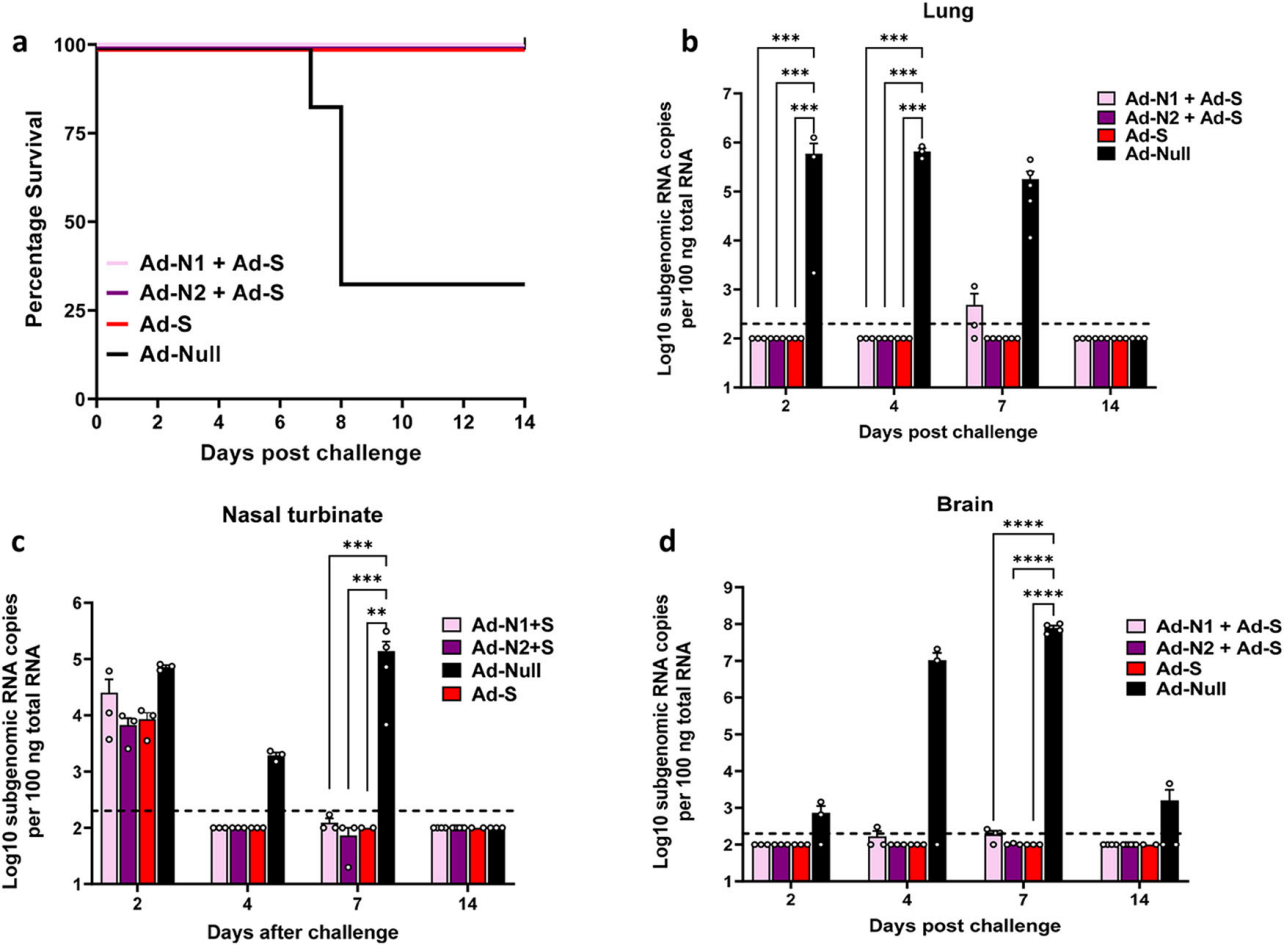
Survival and sgRNA levels in Ad-N1 + Ad-S and Ad-N2 + Ad-S immunized mice post SARS-CoV-2 challenge. **a** Kaplan–Meier survival curve of SARS-CoV-2-challenged K18-hACE2 mice. Subgenomic viral RNA (sgRNA) levels in the lungs (**b**), nasal turbinate (**c**), and brains (**d**) of challenged mice. sgRNA copy numbers falling below the limit of detection (LOD) (200 copies), indicated by the dashed line, were set as half of the LOD. Error bars represent SEM, each dot represents an individual mouse. Statistical analysis was conducted using two-way ANOVA with Tukey’s Multiple Comparisons. Significance levels are indicated as follows: **p < 0.01, ***p < 0.001, ****p < 0.0001. For clarity, comparisons that were not significant are not shown.

We next assessed the protective effect of S plus N immunization compared with S immunization alone in challenged mice. As for the studies using N alone, K18-hACE2 mice were challenged 6 weeks after the 2nd dose of vaccine with 5×10^4^ PFU SARS-CoV-2 (Washington strain, WA1/2020 (614D)) expressing N and S antigens homologous to the proteins encoded by the Ad-N2 and Ad-S recombinants (Fig. 1a). We observed 100% survival in animals that received Ad-S, whether alone or in combination with Ad-N1 or Ad-N2 (Fig. 7a). We were unable to detect sgRNA in the lungs of immunized mice except for two mice in the Ad-N1+Ad-S group on day 7 (Fig. 7b). For all immunized groups lung, nasal turbinate and brain sgRNA titers were lower than in the control Ad-Null group post-challenge (Fig. 7b–d) and at most time points were undetectable in the immunized mice.

**Fig. 8:**
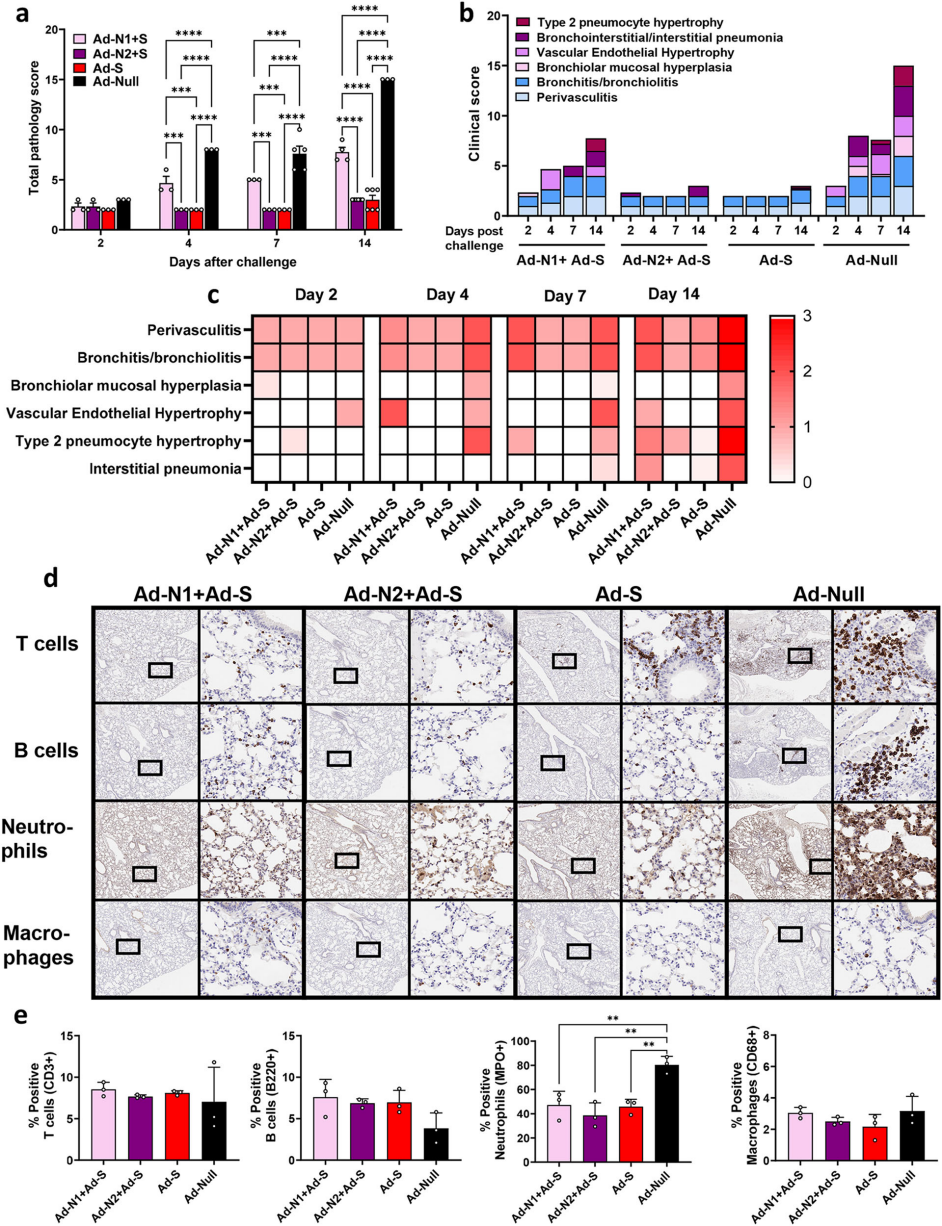
Histopathology analysis of Ad-N1 + Ad-S and Ad-N2 + Ad-S immunized mice following SARS-CoV-2 challenge. H&E staining was performed on formalin-fixed paraffin-embedded lung tissues of mice in each group on specified days after challenge. **a** Mean total pathology score for individual groups. The combined scores of six parameters for lung pathology are presented **b** Mean inflammatory scores for each of six lung pathology parameters for individual groups. **c** Heatmap showing inflammatory levels in immunized mice post-challenge. **d** Immunohistochemistry staining for T cells, B cells, neutrophils and macrophages in lungs of immunized mice at 7 days post-challenge. Images are shown at low (2x) and high (20x) magnification. The box in the low-magnification image represents the area shown in the high-magnification image. **e** Percentage of positive T cells, B cells, neutrophils and macrophages in total scanned lung tissues. Error bars represent SEM. Statistical analysis was conducted using two-way ANOVA (**a**) or one-way ANOVA (**e**) with Tukey’s Multiple Comparisons. Significance levels are indicated as follows: **p < 0.01, ***p < 0.001, ****p < 0.0001. For clarity, comparisons that were not significant are not shown.

Inflammation and pathological scores were also assessed in H&E-stained lung tissue (Fig. 8a). The Ad-S immunized group exhibited very mild pathology progression, including a low degree of perivasculitis and bronchitis throughout, with minimal type 2 pneumocyte hypertrophy and interstitial pneumonia observed only at day 14 (Fig. 8b, c). The addition of Ad-N2 to Ad-S did not impact pathology scores, however, the inclusion of Ad-N1 with Ad-S resulted in increased pathology scores (Fig. 8a) and perivasculitis and bronchitis on all days post-challenge compared to the Ad-N2+Ad-S and Ad-S only groups (Fig. 8b, c and Supplementary Fig. 6). The Ad-N1+Ad-S group exhibited increased cellularity (hyperplasia/hypertrophy) and pneumonia progression, although the degree of cellularity increase and pneumonia progression was lower than that in the Ad-Null group (Fig. 8b, c). Of interest, the pathology observed following Ad-N1+Ad-S immunization was higher than that observed for Ad-N1 alone (Fig. 4). Immunohistochemistry staining of lung tissues on day 7 showed fewer T and B cell clusters in all groups compared to the Ad-Null group (Fig. 8d), as seen for animals immunized with N alone (Fig. 4), although we observed an overall higher frequency of T cells in the lungs of immunized mice (Fig. 8e) compared with the levels observed for the mice immunized with N alone (Fig. 4e). We also observed slightly higher total B cell numbers in the scanned tissue sections from the immunized mice (Fig. 8e). In contrast to the mice immunized with N antigen alone (Fig. 4), we observed lower levels of neutrophil infiltration in the lung tissues of immunized mice compared to the control mice (Fig. 8d, e) while observing similar low levels of macrophages in all groups (Fig. 8d, e).

## Discussion

In this study, we evaluated the immunogenicity and protective efficacy of adenovirus constructs expressing the nucleocapsid (N) of SARS-CoV (Ad-N1) or SARS-CoV-2 (Ad-N2) either alone, i.e. in the absence of a neutralizing antibody response, or in combination with an adenovirus construct expressing the SARS-CoV-2 spike (S) protein (Ad-S) using K18-hACE2 mice. T cells have been shown to play important roles in protection from many viral infections. A recent publication emphasized the potential for S-specific T cells to play a role in antibody-independent protection^20^. The inclusion of a more conserved antigen such as N can address the issue of S antigen variation while at the same time providing added T cell protection. However, previous publications have shown enhanced lung pathology following challenge in mice immunized with the N and S antigens of SARS-CoV^26^. In addition, there have been case reports of enhanced lung pathology in COVID-19 patients treated with convalescent plasma^30,31^. Although convalescent plasma transfusions have been safely used for treatment in many COVID-19 patients without enhanced disease^32,33^, these individual cases underscore the importance of understanding immune system reactions that can lead to adverse outcomes. Although enhanced disease outcomes in animal models have been associated with a Th2 type response for SARS-CoV-2^34,35^, there remain concerns regarding the safety of combined immunization, particularly in cases where there is a mismatch between the immunizing antigen and the infecting virus. We assessed this potential of T cell responses to provide protective immune responses against variant viruses by inducing N-specific T cell responses to SARS-CoV and SARS-CoV-2 followed by challenge with SARS-CoV-2 in K18-hACE2 mice and the impact of including the S antigen in the immunization regimen.

The immunized mice developed robust humoral and cellular immune responses against all antigens, characterized by high titers of SARS-CoV-2 N and S binding antibodies, the induction of antigen-specific T cell responses, and the induction of neutralizing antibodies in mice immunized with Ad-S. When administered alone, Ad-N1 and Ad-N2 induced immune responses that provided low-level protection against SARS-CoV-2 challenge, with slightly improved survival rates and significantly reduced viral sgRNA titers and pathology in the lungs compared to the control immunized group (Figs. 3 and 4). We used sgRNA to assess active replication of SARS-CoV-2 in tissues, this has been shown to be a reliable indicator of replicating virus^36^. The low-level protection that we observed in N immunized mice is consistent with previous studies showing that N can induce protective immunity in preclinical models^21–25^. There were no differences in sgRNA titers in the brain and nasal turbinates of any mice post challenge following immunization with N antigen alone (Fig. 3). This low-level control of virus replication is consistent with the absence of neutralizing antibodies, which are necessary to prevent viral infection. The K18-hACE2 model is useful for studying vaccine effectiveness and pathology following SARS-CoV-2 infection, but the virus can spread to the brain, resulting in encephalitis and poor survival, hence the detection of sgRNA in the brains of challenged mice^37,38^ (Fig. 3). Despite significantly lower levels of sgRNA in the lungs of both Ad-N1 and Ad-N2 immunized mice early during infection (days 2 and 4 post challenge) (Fig. 3), at day 7 sgRNA titers in the lungs of mice immunized with Ad-N1 were not lower than those in the Ad-Null group and higher than those in the Ad-N2 group (Fig. 3). This suggests that the control of viral replication in the Ad-N1 immunized mice was less effective than the control in the Ad-N2 group. Notably, lung pathology assessments revealed a considerable decrease in inflammatory infiltration, perivasculitis, and bronchitis in the N immunized groups compared to the control group (Fig. 3). The milder pulmonary pathology progression observed in the N-immunized groups indicated that the induced T cells functioned in lungs effectively to control viral replication resulting in a mitigation of lung damage and supporting the protective role of N-specific immune responses in SARS-CoV-2 infection. However, consistent with the slightly reduced control of virus replication post challenge in the Ad-N1 group compared to the Ad-N2 group, we observed slightly increased pathology, although not statistically significant, in the Ad-N1 group (Fig. 4) compared to the Ad-N2 group post challenge. This may be associated with the detection of higher levels of CD8 + T cells expressing the cytokines IFN-γ, TNF-α and cytotoxicity markers (granzyme B and perforin) in the Ad-N1 immunized mice following stimulation of spleen cells with peptides corresponding to the SARS-CoV-2 N antigen (Figs. 1 and 2). The reason for the higher T cell responses in the Ad-N1 immunized mice is not clear. This could be due to better processing of the N1 antigen due to the amino acid differences in the sequence, which lead to enhanced presentation of the antigenic epitopes to the immune cells. Additional studies would be required to fully understand this outcome. Upon infection with virus CD8+ T cells kill infected cells and produce cytokines and inflammatory molecules, which can result in the recruitment of additional immune cells to the site of viral replication. We observed some very high responders in the T cell studies in the Ad-N1 group (Fig. 2a–c). When data from these animals were removed from the analysis, significance was maintained for some comparisons. When significance was not maintained, the mean responses for the Ad-N1 immunized group remained higher than those in the Ad-N2 immunized group, supporting the conclusion that immunization with Ad-N1 resulted in higher T cell responses in the K18 mice. One explanation for our observations of increased pathology in the N1 immunized mice is that the stronger T cell immune responses induce by N1, evidenced from the in vitro studies, resulted in increased T cell activation following challenge with SARS-CoV-2, leading to increased production of cytokines and inflammatory molecules, which in turn led to increased pathology compared to the N2 immunized mice. Interestingly, the in vitro CD4+ response was found to be significantly higher in the Ad-N2 group compared with the Ad-N1 group (Fig. 2), which could have resulted in a better T helper response post-challenge in the Ad-N2 immunized mice leading to better control of virus replication.

Combining immunization against N1 or N2 with immunization against the S antigen of SARS-CoV-2 successfully induced immune responses to both antigens (Fig. 5, 6). The CD8+ T cell responses to S in the Ad-N1+Ad-S and Ad-N2+Ad-S groups were consistently higher than in the Ad-S alone group, suggesting a stimulatory effect of the N immunization for T cell responses against S. The N antigen immunization may increase the recruitment of antigen-presenting cells, higher cytokine production or increased T helper activity which in turn could enhance the T cell immune response to the S antigen. We also observed higher levels of S antigen-binding antibodies in the combined groups compared to the S alone immunized group (Fig. 5), although the opposite was the case for neutralizing antibody titers, with significantly lower titers detected in the groups with combined immunization. This suggests an impact of the N antigen on the presentation of the S antigen to the immune system or interference from the anti-N antibodies in the neutralization reaction. Further studies on epitope recognition and possible interference from N-specific antibodies on S neutralizing antibodies would be interesting and important for assessing future combination vaccines. Despite the reduced levels of neutralizing antibodies in the N + S groups, all animals immunized with Ad-S exhibited 100% survival, significantly lower lung viral titers, and milder pathology compared to the control group. However, the addition of Ad-N1 to Ad-S resulted in increased pathology compared to the groups immunized with Ad-N2+Ad-S or Ad-S alone, albeit at a lower level than the Ad-Null group (Fig. 8). This is consistent with our observations in N-only immunized mice following challenge (Fig. 4) and could also be explained by the higher T cell responses in the mice receiving Ad-N1 in combinations with Ad-S resulting in increased T cell activation following challenge.

The higher levels of T cells detected ex vivo in the Ad-N1 immunized mice did not correlate with higher T cell infiltration in the lungs post challenge (Figs. 4, 8). However, the inclusion of S antigen in the vaccine resulted in higher T and B cell infiltration and lower neutrophil infiltration compared with N immunization alone. This is consistent with the role of neutrophils in innate immunity such that there is a higher level of recruitment of these cells to the lungs in the absence of neutralizing antibodies in the N-only immunized mice and a more effective adaptive immune response from the combined immunization.

Previously, Dangi et al.^24^ showed that the addition of N antigen to S for immunization of K18-hACE2 mice was necessary to improve control of SARS-CoV-2 infection in the brain. We did not find this to be the case. In contrast, we found that inclusion of the S antigen for immunization was required to prevent dissemination of the virus to the brain. Viral titers in the lungs and brains of challenged mice that were immunized with S antigen, either alone or in combination with N, were below the limit of detection for all immunized groups, with the exception of individual samples from certain time points for mice immunized with Ad-N1+Ad-S. Our data suggest a slight inhibitory effect of N1 on S immune responses. Our studies and those of Dangi et al. used adenovirus recombinants for immunization. The differences in outcomes could be due to the specific sequences used, the use of a prime/boost regimen in our studies or the specific virus used for challenge.

Overall, our findings suggest that nucleocapsid antigen induces strong immune responses and that even a mismatched antigen confers a degree of protection against SARS-CoV-2 challenge in K18-hACE2 mice. The combination of N and S antigens did not compromise the protective effects but there appeared to be a mildly negative impact of N on neutralizing antibody responses induced by S and an increase in inflammatory responses in mice co-immunized with N1 compared to S alone. It should be noted that these observations have been made in the K18-hACE2 transgenic mouse model and the increased pathology with the N antigen from SARS-CoV may not occur in human infections, although increased T cell immunity arising from combined S and N immunization could feasibly lead to increased cytokine production and inflammation associated with protection in humans. These outcomes may also be specific to the N antigen from SARS-CoV and variants of SARS-CoV-2 may not result in enhanced pathology. However, even if this were to be the case for any other N antigen variant, such outcomes may be acceptable if the combination of N and S in a vaccine results in increased breadth of protection in the population.

## Methods

### Adenovirus SARS-CoV and SARS-CoV-2 recombinants

Recombinant nonreplicating adenovirus vectors expressing the nucleocapsid of SARS-CoV Urbani isolate (GenBank Accession number MK062184.1) (Ad-N1), the nucleocapsid of SARS-CoV-2 (Ad-N2) or the spike protein of SARS-CoV-2 (SARS-CoV-2/human/USA/WA2/2020 (GenBank Accession number MT152824) (Ad-S) were constructed using the AdEasy XL Adenoviral vector system according to the manufacturer’s instructions (Agilent, CA).

### SARS-CoV-2 pseudotyped viruses (SARSpp) neutralization assay

Lentivirus-based SARS-CoV-2 S pseudotyped viruses were prepared as previously described^39,40^. The neutralization assay was performed according to the protocol described previously^40^ using the 293T-ACE2/TMPRSS2 cell line^41^ Results for each well were obtained as RLU, and 50% inhibitory dilution (ID50) neutralization titers were calculated using the Reed and Muench method. Titers were expressed as a reciprocal of the dilution calculated to neutralize 50% of the RLU signal. A titer of 100 was assigned to samples when <50% inhibition was obtained at 1:200 dilution.

### Animals, immunization, and challenge

Animal experiments were performed using 6–8-week-old K18-Tg hACE2 (K18-hACE2) mice (#034860, Jackson Labs). All immunizations, tissue harvests and blood draws were performed in accordance with an animal protocol approved by the FDA White Oak Consolidated Animal Program (Protocol #2020-09). Blood draws (50 µl) were performed via the submandibular route, spleens were collected from mice euthanized by CO_2_ overdose followed by cervical dislocation. For all infection and immunization studies mice were anesthetized by 3–5% isoflurane inhalation. Intramuscular inoculations into the hind quadriceps muscle of 1 × 10^9^ pfu of adenovirus recombinants diluted in PBS (50 µl) were performed at 0 and 28 days (Fig. 1a). For infection studies mice were inoculated intranasally using 5 × 10^4^ PFU SARS-CoV-2 (USA/WA1/2020) (25 µl) 70 days after the primary inoculation. Lung, brain, and nasal turbinate tissues for histopathologic evaluation and virus-titer analysis were collected from euthanized mice upon morbidity or on days 2, 4, 7 and 14 post-challenge (Fig. 1a). Mice were euthanized by CO_2_ overdose followed by cervical dislocation.

### RNA isolation from tissues and RT-PCR assay of SARS-CoV-2 subgenomic RNA

RNA was extracted from tissues using Trizol (15-596-018, Fisher Scientific) according to the manufacturer’s instructions and resuspended in 50 μL of DEPC-treated water. Quantification of SARS-CoV-2 subgenomic and genomic RNA was performed as described previously^18^.

### Lung Histopathology and inflammation scores, immunochemical staining

Formalin-fixed paraffin-embedded lung tissues of mice were hematoxylin and eosin (H&E) or immunohistochemically (IHC) stained by Histoserv, Inc (Germantown, MD, 394 USA). Slides were scanned using an Aperio ImageScope. Histopathological scores were assigned using the following characteristics: perivasculitis, bronchitis, bronchiolar mucosal hyperplasia, vascular endothelial hypertrophy, interstitial pneumonia, and type 2 pneumocyte hypertrophy^42^. The following antibodies were used for IHC staining: goat anti-NKp46/NCR1 antibody (NK cells) (AF2225, R&D), rabbit anti-CD68 (macrophages) (#125212, Abcam), rabbit anti-MPO (neutrophils)(AF3667, R&D), rat anti-CD45R/B220 (B cells) (#14-0452-82 ThermoFisher), rabbit anti-CD3e (78588, Cell Signaling Technology). The open-source software QuPath^43^ was utilized for bioimage analysis to determine the percentage of positive cells in IHC-stained lung sections.

### Enzyme-linked immunosorbent assay (ELISA)

To detect binding antibodies to SARS-CoV2 nucleocapsid or spike protein in sera, plates were coated overnight at 4°C with recombinant nucleocapsid protein or spike protein (1 µg/mL) as previously described^40^. Serum samples were serially diluted 2-fold starting at a 1:100 dilution. Endpoint titers were determined using a positive to negative (P/N) OD405 ratio less than 2. To calculate the P/N ratio, naïve mouse serum was diluted in parallel with the test samples. All samples were tested in duplicate. The mean OD signals for negative controls and test samples were calculated at each dilution and a P/N ratio was determined by dividing the test sample OD value by the OD value for the negative control. A P/N value ≥ 2 was considered positive. All samples with a P/N value less than 2 were considered negative.

### Splenocytes isolation and IFN-γ ELISpot assay

Spleen cells were isolated two weeks post boost and the IFN-γ production upon in vitro stimulation was analyzed using mouse IFN-γ ELISpot kits (catalog number 3321-2H, Mabtech) according to the manufacturer’s instructions using overlapping peptide pools (2 μg/mL per peptide) peptide from a peptide array corresponding to the full length SARS-CoV-2 nucleocapsid protein (BEI Resources NR-52404) or the full length SARS-CoV-2 spike protein (BEI Resources NR-52402). Single nucleocapsid peptide N220 was used at a final concentration of 5 μg/ml, Concanavalin A (ConA) was used as a positive control (2 μg/ml), and a pool of Ebola virus glycoprotein peptides was used as negative control (2 μg/mL per peptide). Plates were incubated at 37°C, 5% CO_2_ for 40 to 44 h. Spots were revealed using biotinylated detection Ab (R4-6A2), Strepta-HRP Ab, and TMB ELISpot substrate and counted using an ImmunoSpot plate analyzer (Cellular Technology Limited).

### Flow cytometry analysis

Splenocytes (5×10^5^ cells) were seeded in round bottom 96 well plates and stimulated by addition of SARS-CoV-2 nucleocapsid or spike peptide pools (2 μg/ml each peptide), N220 peptide (5 μg/mL), positive control (ConA, 2 μg/ml), or a pool of Ebola virus glycoprotein peptides as a negative control (2 μg/mL per peptide). Cells were stimulated for total of 24 h. Brefeldin A (5 μg/mL) and monensin (2 μM) were added to block cytokine secretion 4 hours before collection. Stimulated cells were stained for surface antigens then permeabilized with Fix/Perm buffer (BD Bioscience). Cellular events were acquired on a BD FACS Fortessa multi-parameter flow cytometer (BD). The following antibodies were used: CD44 (#103059), CD4 (#100430), Perforin (#154304), IL-4 (#504109), Granzyme B (#372216), IL-2 (#503808) TNF-α (#506324), CD19 (#115540), IFN-γ (#505836), CD3e (#100222) (Biolegend, San Diego, CA), CD8a (#566409) (BD Biosciences, San Jose, CA), fixable live-dead near IR (ThermoFisher, Grand Island, NY). The gating strategy used for analysis is shown in Supplementary Fig. 7.

### Data and statistical analysis

Flow cytometry data were analyzed with FlowJo software (10.4) (Tree Star, OR). Statistical analyses were performed by using a one-way or two-way analysis of variance (ANOVA) in Graphpad software to compare means. When a statistically significant difference between the means was determined using one-way ANOVA a post hoc analysis was used to assess differences between the means of each group. A *p* value of < 0.05 was considered significant. Significance levels are indicated as follows: *p < 0.05, **p < 0.01, ***p < 0.001, ****p < 0.0001. For clarity, comparisons that were not significant are not shown.

## Supplementary information

Supplementary Materials

## Data Availability

This study does not contain any datasets and the study did not generate any new sequence data. Recombinant viral vectors were generated from existing sequences, the Genbank Accession numbers are MK062184.1 and MT152824. The data generated during this study are available from the corresponding author on reasonable request.

## References

[1] Coronaviridae Study Group of the International Committee on Taxonomy of, V. The species Severe acute respiratory syndrome-related coronavirus: classifying 2019-nCoV and naming it SARS-CoV-2. *Nat. Microbiol.* **5**, 536–544 (2020). 10.1038/s41564-020-0695-z PMC7095448 32123347

[2] WHO_COVID19_dashboard. *WHO Coronavirus Disease (COVID-19) Dashboard*, https://covid19.who.int (2021).

[3] Baden, L. R. et al. Efficacy and safety of the mRNA-1273 SARS-CoV-2 vaccine. *N. Engl. J. Med.* **384**, 403–416 (2021). 33378609 10.1056/NEJMoa2035389 PMC7787219

[4] Polack, F. P. et al. Safety and efficacy of the BNT162b2 mRNA Covid-19 vaccine. *N. Engl. J. Med.* **383**, 2603–2615 (2020). 33301246 10.1056/NEJMoa2034577 PMC7745181

[5] Yang, Z. R. et al. Efficacy of SARS-CoV-2 vaccines and the dose-response relationship with three major antibodies: a systematic review and meta-analysis of randomised controlled trials. *Lancet Microbe* **4**, e236–e246 (2023). 36868258 10.1016/S2666-5247(22)00390-1 PMC9974155

[6] Cao, Y. et al. Omicron escapes the majority of existing SARS-CoV-2 neutralizing antibodies. *Nature* **602**, 657–663 (2022). 35016194 10.1038/s41586-021-04385-3 PMC8866119

[7] Jian, F. et al. Further humoral immunity evasion of emerging SARS-CoV-2 BA.4 and BA.5 subvariants. *Lancet Infect. Dis.* **22**, 1535–1537 (2022). 36179744 10.1016/S1473-3099(22)00642-9 PMC9514837

[8] Pather, S. et al. SARS-CoV-2 Omicron variants: burden of disease, impact on vaccine effectiveness and need for variant-adapted vaccines. *Front. Immunol.* **14**, 1130539 (2023). 37287979 10.3389/fimmu.2023.1130539 PMC10242031

[9] Snijder, E. J. et al. Unique and conserved features of genome and proteome of SARS-coronavirus, an early split-off from the coronavirus group 2 lineage. *J. Mol. Biol.* **331**, 991–1004 (2003). 12927536 10.1016/S0022-2836(03)00865-9 PMC7159028

[10] McBride, R., van Zyl, M. & Fielding, B. C. The coronavirus nucleocapsid is a multifunctional protein. *Viruses* **6**, 2991–3018 (2014). 25105276 10.3390/v6082991 PMC4147684

[11] He, Y. et al. Mapping of antigenic sites on the nucleocapsid protein of the severe acute respiratory syndrome coronavirus. *J. Clin. Microbiol* **42**, 5309–5314 (2004). 15528730 10.1128/JCM.42.11.5309-5314.2004 PMC525273

[12] Meyer, B., Drosten, C. & Muller, M. A. Serological assays for emerging coronaviruses: challenges and pitfalls. *Virus Res.* **194**, 175–183 (2014). 24670324 10.1016/j.virusres.2014.03.018 PMC7114385

[13] Dutta, N. K., Mazumdar, K. & Gordy, J. T. The nucleocapsid protein of SARS-CoV-2: a target for vaccine development. *J. Virol.* **94**, e00647–20 (2020). 32546606 10.1128/JVI.00647-20 PMC7307180

[14] Lopez-Munoz, A. D., Kosik, I., Holly, J. & Yewdell, J. W. Cell surface SARS-CoV-2 nucleocapsid protein modulates innate and adaptive immunity. *Sci. Adv.* **8**, eabp9770 (2022). 35921414 10.1126/sciadv.abp9770 PMC9348789

[15] Grifoni, A. et al. SARS-CoV-2 human T cell epitopes: Adaptive immune response against COVID-19. *Cell Host Microbe* **29**, 1076–1092 (2021). 34237248 10.1016/j.chom.2021.05.010 PMC8139264

[16] Mateus, J. et al. Low-dose mRNA-1273 COVID-19 vaccine generates durable memory enhanced by cross-reactive T cells. *Science* **374**, eabj9853 (2021). 34519540 10.1126/science.abj9853 PMC8542617

[17] Goel, R. R. et al. mRNA vaccines induce durable immune memory to SARS-CoV-2 and variants of concern. *Science* **374**, abm0829 (2021). 34648302 10.1126/science.abm0829 PMC9284784

[18] Liu, S. et al. Intranasal delivery of a rationally attenuated SARS-CoV-2 is immunogenic and protective in Syrian hamsters. *Nat. Commun.* **13**, 6792 (2022). 36357440 10.1038/s41467-022-34571-4 PMC9648440

[19] Wherry, E. J. & Barouch, D. H. T cell immunity to COVID-19 vaccines. *Science* **377**, 821–822 (2022). 35981045 10.1126/science.add2897

[20] Fumagalli, V. et al. Antibody-independent protection against heterologous SARS-CoV-2 challenge conferred by prior infection or vaccination. *Nat. Immunol.* **25**, 633–643 (2024). 38486021 10.1038/s41590-024-01787-z PMC11003867

[21] Hajnik, R. L. et al. Dual spike and nucleocapsid mRNA vaccination confer protection against SARS-CoV-2 Omicron and Delta variants in preclinical models. *Sci. Transl. Med.* **14**, eabq1945 (2022). 36103514 10.1126/scitranslmed.abq1945 PMC9926941

[22] Primard, C. et al. OVX033, a nucleocapsid-based vaccine candidate, provides broad-spectrum protection against SARS-CoV-2 variants in a hamster challenge model. *Front. Immunol.* **14**, 1188605 (2023). 37409116 10.3389/fimmu.2023.1188605 PMC10319154

[23] Arieta, C. M. et al. The T-cell-directed vaccine BNT162b4 encoding conserved non-spike antigens protects animals from severe SARS-CoV-2 infection. *Cell* **186**, 2392–2409.e2321 (2023). 37164012 10.1016/j.cell.2023.04.007 PMC10099181

[24] Dangi, T., Class, J., Palacio, N., Richner, J. M. & Penaloza MacMaster, P. Combining spike- and nucleocapsid-based vaccines improves distal control of SARS-CoV-2. *Cell Rep.* **36**, 109664 (2021). 34450033 10.1016/j.celrep.2021.109664 PMC8367759

[25] Rak, A., Isakova-Sivak, I. & Rudenko, L. Overview of nucleocapsid-targeting vaccines against COVID-19. *Vaccines (Basel)* **11**, 1810 (2023). 38140214 10.3390/vaccines11121810 PMC10747980

[26] Yasui, F. et al. Prior immunization with severe acute respiratory syndrome (SARS)-associated coronavirus (SARS-CoV) nucleocapsid protein causes severe pneumonia in mice infected with SARS-CoV. *J. Immunol.* **181**, 6337–6348 (2008). 18941225 10.4049/jimmunol.181.9.6337

[27] Rochman, N. D. et al. Ongoing global and regional adaptive evolution of SARS-CoV-2. *Proc. Natl Acad. Sci. USA* **118**, e2104241118 (2021). 34292871 10.1073/pnas.2104241118 PMC8307621

[28] N’Guessan, A. et al. Selection for immune evasion in SARS-CoV-2 revealed by high-resolution epitope mapping and sequence analysis. *iScience* **26**, 107394 (2023). 37599818 10.1016/j.isci.2023.107394 PMC10433132

[29] Joag, V. et al. Cutting edge: mouse SARS-CoV-2 epitope reveals infection and vaccine-elicited CD8 T cell responses. *J. Immunol.* **206**, 931–935 (2021). 33441437 10.4049/jimmunol.2001400 PMC8136468

[30] Amrutiya, V. et al. Transfusion-related acute lung injury in a COVID-19-positive convalescent plasma recipient: a case report. *J. Int. Med. Res.* **49**, 3000605211032814 (2021). 34412545 10.1177/03000605211032814 PMC8381434

[31] Wang, K. Y., Shah, P. & Pierce, M. Convalescent plasma for COVID-19 complicated by ARDS due to TRALI. *BMJ Case Rep.* **14**, e239762 (2021). 33509890 10.1136/bcr-2020-239762 PMC10577747

[32] Bussani, R. et al. Lung damage in SARS-CoV-2 patients: An autopsy study in the era of vaccination. *Eur. J. Clin. Invest.* **55**, e14325 (2025). 39344023 10.1111/eci.14325 PMC11628649

[33] Duan, K. et al. Effectiveness of convalescent plasma therapy in severe COVID-19 patients. *Proc. Natl Acad. Sci. USA* **117**, 9490–9496 (2020). 32253318 10.1073/pnas.2004168117 PMC7196837

[34] Iwata-Yoshikawa, N. et al. A lethal mouse model for evaluating vaccine-associated enhanced respiratory disease during SARS-CoV-2 infection. *Sci. Adv.* **8**, eabh3827 (2022). 34995117 10.1126/sciadv.abh3827 PMC8741184

[35] Ebenig, A. et al. Vaccine-associated enhanced respiratory pathology in COVID-19 hamsters after T(H)2-biased immunization. *Cell Rep.* **40**, 111214 (2022). 35952673 10.1016/j.celrep.2022.111214 PMC9346010

[36] Zhang, C. et al. SARS-CoV-2 virus culture, genomic and subgenomic RNA load, and rapid antigen test in experimentally infected Syrian hamsters. *J. Virol.* **96**, e0103422 (2022). 36040179 10.1128/jvi.01034-22 PMC9517720

[37] McCray, P. B. Jr et al. Lethal infection of K18-hACE2 mice infected with severe acute respiratory syndrome coronavirus. *J. Virol.* **81**, 813–821 (2007). 17079315 10.1128/JVI.02012-06 PMC1797474

[38] Oladunni, F. S. et al. Lethality of SARS-CoV-2 infection in K18 human angiotensin-converting enzyme 2 transgenic mice. *Nat. Commun.* **11**, 6122 (2020). 33257679 10.1038/s41467-020-19891-7 PMC7705712

[39] Korber, B. et al. Tracking changes in SARS-CoV-2 spike: evidence that D614G increases infectivity of the COVID-19 virus. *Cell* **182**, 812–827.e819 (2020). 32697968 10.1016/j.cell.2020.06.043 PMC7332439

[40] Kachko, A. et al. Vaccine-associated respiratory pathology correlates with viral clearance and protective immunity after immunization with self-amplifying RNA expressing the spike (S) protein of SARS-CoV-2 in mouse models. *Vaccine* **42**, 608–619 (2024). 38142216 10.1016/j.vaccine.2023.12.052

[41] Neerukonda, S. N. et al. Establishment of a well-characterized SARS-CoV-2 lentiviral pseudovirus neutralization assay using 293T cells with stable expression of ACE2 and TMPRSS2. *PLoS ONE* **16**, e0248348 (2021). 33690649 10.1371/journal.pone.0248348 PMC7946320

[42] Winarski, K. L. et al. Antibody-dependent enhancement of influenza disease promoted by increase in hemagglutinin stem flexibility and virus fusion kinetics. *Proc. Natl Acad. Sci. USA* **116**, 15194–15199 (2019). 31296560 10.1073/pnas.1821317116 PMC6660725

[43] Bankhead, P. et al. QuPath: Open source software for digital pathology image analysis. *Sci. Rep.* **7**, 16878 (2017). 29203879 10.1038/s41598-017-17204-5 PMC5715110

