# Supplementary material for: Combined immunization with SARS-CoV-2 spike and SARS-CoV nucleocapsid protects K18-hACE2 mice but increases lung pathology
Source: NPJ Vaccines. 2025 Feb 13;10:30. doi: 10.1038/s41541-025-01085-1 (PMC11825953; doi:10.1038/s41541-025-01085-1)
Supplement: Supplementary file 1 — Supplementary Materials [file 41541_2025_1085_MOESM1_ESM.pdf]

WHO label	Pango lineage	GenBank reference	Total number of amino acids in nucleocapsid	Number of amino acid differences in nucleocapsid	% difference
**SARS-CoV***		MK062184.1	422	41	9.8
**WA2/2020***		MT152824	419	0	0.00
**Alpha**	B.1.1.7	OR584241.1	419	4	0.95
**Beta**	B.1.351	OM366051.1	419	0	0.00
**Gamma**	P.1	OR578388.1	419	0	0.00
**Epsilon**	B.1.429	OR322718.1	419	0	0.00
**Eta**	B.1.525	MZ362451.1	418	4	0.95
**Mu**	B.1.621	OQ248293.1	419	2	0.5
**Delta**	B.1.617.2	OK091006.1	419	4	0.95
**Lambda**	C.37	MZ275302.1	419	5	1.2
**Omicron**	BA.1	OR575560.1	416	6	1.4
**Omicron**	BA.2	ON545852.1	416	7	1.7
**Omicron**	BA.4	OP093374.1	416	8	1.9
**Omicron**	BA.5	OP164785.1	416	7	1.7
**Omicron**	XBB.1.9.1	OR485908.1	419	4	0.95
**Omicron**	JN.1	PP357841.1	416	8	1.9

* Not a WHO label

Supplementary Table 1. Amino acid differences between the nucleocapsids of the SARS-CoV or SARS-CoV-2 variants compared to the SARS-CoV-2 Wuhan strain.

```
N1 MSDNGPQSNQRSAPRITFGGPTDSTDNNQNGGRNGARPKQRRPQGLPNNTASWFTALTQH
N2 MSDNGPQ-NQRNAPRITFGGPSDSTGSNQNGERSGARSKQRRPQGLPNNTASWFTALTQH
   ******* ***.*********:***..**** *.***.**********************

N1 GKEELRFPRGQGVPINTNSGPDDQIGYYRRATRRVRGGDGKMKELSPRWYFYYLGTGPEA
N2 GKEDLKFPRGQGVPINTNSSPDDQIGYYRRATRRIRGGDGKMKDLSPRWYFYYLGTGPEA
   ***:*:*************.**************:********:****************

N1 SLPYGANKEGIVWVATEGALNTPKDHIGTRNPNNNAATVLQLPQGTTLPKGFYAEGSRGG
N2 GLPYGANKDGIIWVATEGALNTPKDHIGTRNPANNAAIVLQLPQGTTLPKGFYAEGSRGG
   .*******:**:******************** **** **********************

N1 SQASSRSSSRSRGNSRNSTPGSSRGNSPARMASGGGETALALLLLDRLNQLESKVSGKGQ
N2 SQASSRSSSRSRNSSRNSTPGSSRGTSPARMAGNGGDAALALLLLDRLNQLESKMSGKGQ
   ************..***********.******..**::****************:*****

N1 QQQGQTVTKKSAAEASKKPRQKRTATKQYNVTQAFGRRGPEQTQGNFGDQDLIRQGTDYK
N2 QQQGQTVTKKSAAEASKKPRQKRTATKAYNVTQAFGRRGPEQTQGNFGDQELIRQGTDYK
   *************************** **********************:*********

N1 HWPQIAQFAPSASAFFGMSRIGMEVTPSGTWLTYHGAIKLDDKDPQFKDNVILLNKHIDA
N2 HWPQIAQFAPSASAFFGMSRIGMEVTPSGTWLTYTGAIKLDDKDPNFKDQVILLNKHIDA
   ********************************** **********:***:**********

N1 YKTFPPTEPKKDKKKKTDEAQPLPQRQKKQPTVTLLPAADMDDFSRQLQNSMSGASADST
N2 YKTFPPTEPKKDKKKKADETQALPQRQKKQQTVTLLPAADLDDFSKQLQQSMSSA--DST
   ****************:**:*.******** *********:****:***:***.*.:***

N1 QA
N2 QA
   **
```

Supplementary Figure 1. Comparison of SARS-CoV and SARS-CoV-2 nucleocapsid amino acid sequences. N1 = SARS-CoV nucleocapsid, nucleocapsid (Urbani isolate GenBank Accession number MK062184.1), N2 = SARS-CoV-2 nucleocapsid (SARS-CoV-2/human/USA/WA2/2020 (GenBank Accession number MT152824). Differences are highlighted in blue. Conserved amino acids as shown as an asterisk. The N220 T cell epitope is shown in bold and underlined text.

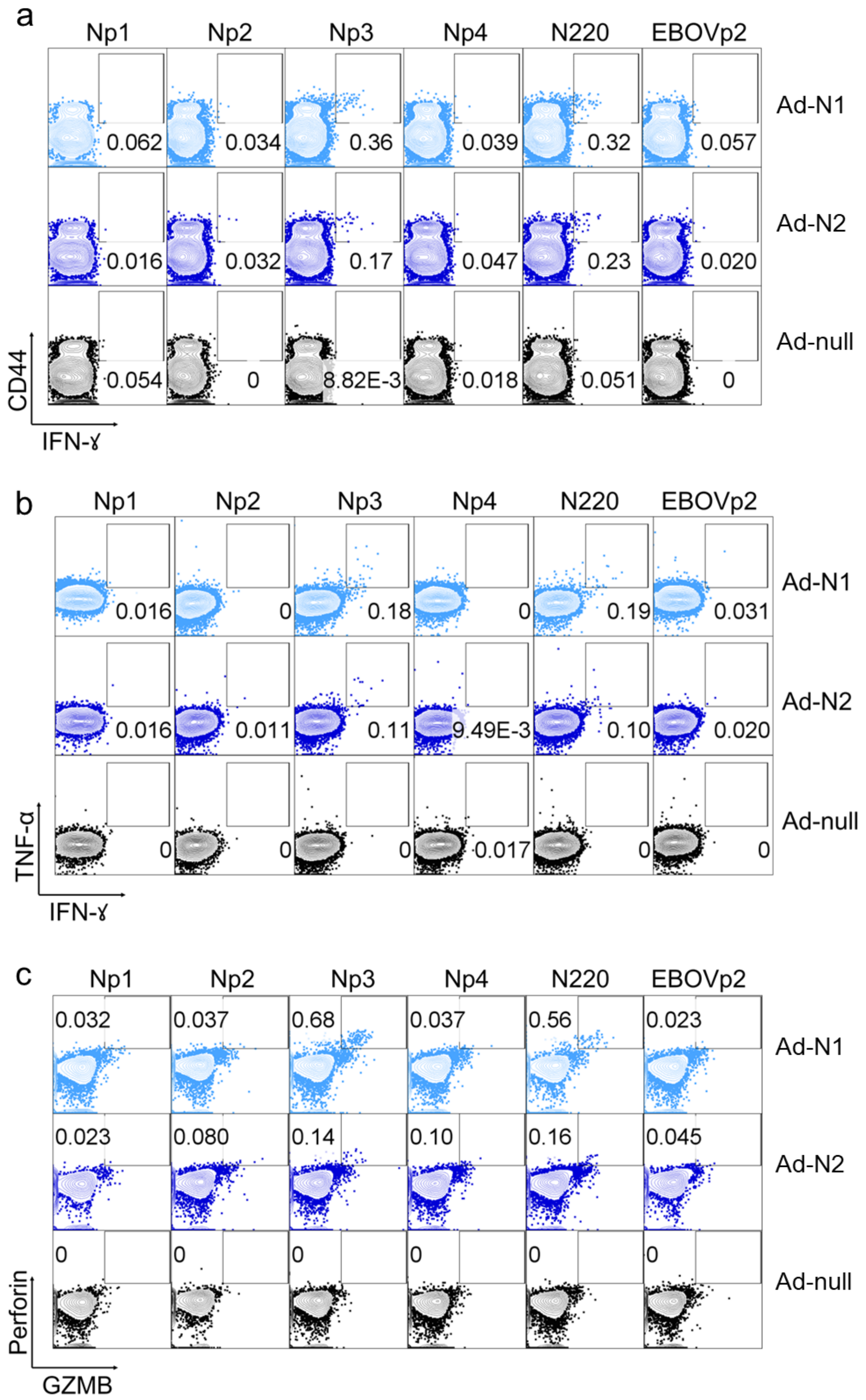
a
Np1
Np2
Np3
Np4
N220
EBOVp2
Ad-N1
0.062
0.034
0.36
0.039
0.32
0.057
Ad-N2
0.016
0.032
0.17
0.047
0.23
0.020
Ad-null
0.054
0
8.82E-3
0.018
0.051
0
CD44
IFN-ɣ
b
Np1
Np2
Np3
Np4
N220
EBOVp2
Ad-N1
0.016
0
0.18
0
0.19
0.031
Ad-N2
0.016
0.011
0.11
9.49E-3
0.10
0.020
Ad-null
0
0
0
0.017
0
0
TNF-α
IFN-ɣ
c
Np1
Np2
Np3
Np4
N220
EBOVp2
Ad-N1
0.032
0.037
0.68
0.037
0.56
0.023
Ad-N2
0.023
0.080
0.14
0.10
0.16
0.045
Ad-null
0
0
0
0
0
0
Perforin
GZMB

d

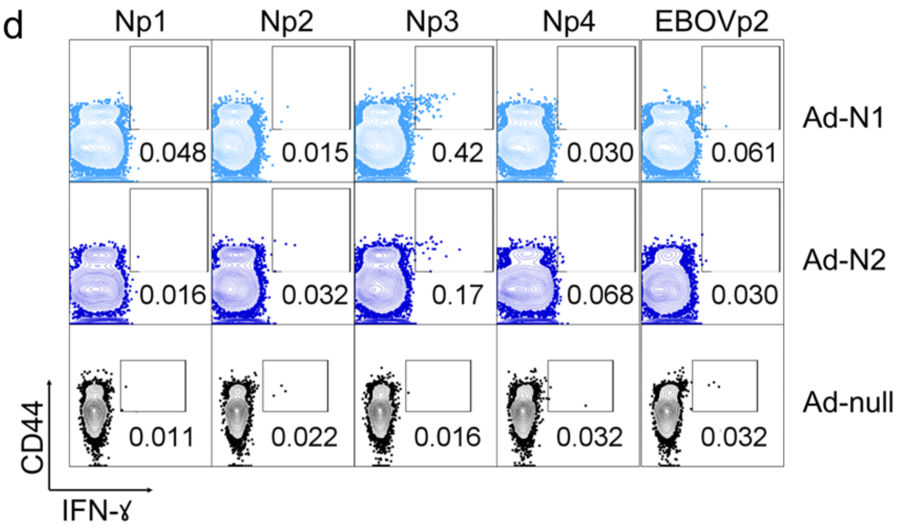

Supplementary Figure 2: Representative flow cytometry dot plots for data shown in Figure 2. Representative flow cytometry dot plots from one mouse per group for data shown in Figures 2a-d. **a**. CD8+ cells expressing IFN-γ following SARS CoV-2 N peptide stimulation. **b**. CD8+ cells expressing both IFN-γ and TNF-α following SARS CoV-2 N peptide stimulation. **c**. CD8+ cells expressing both Granzyme B and Perforin following SARS CoV-2 N peptide stimulation. **d**. CD4+ cells expressing IFN-γ SARS CoV-2 N peptide stimulation.

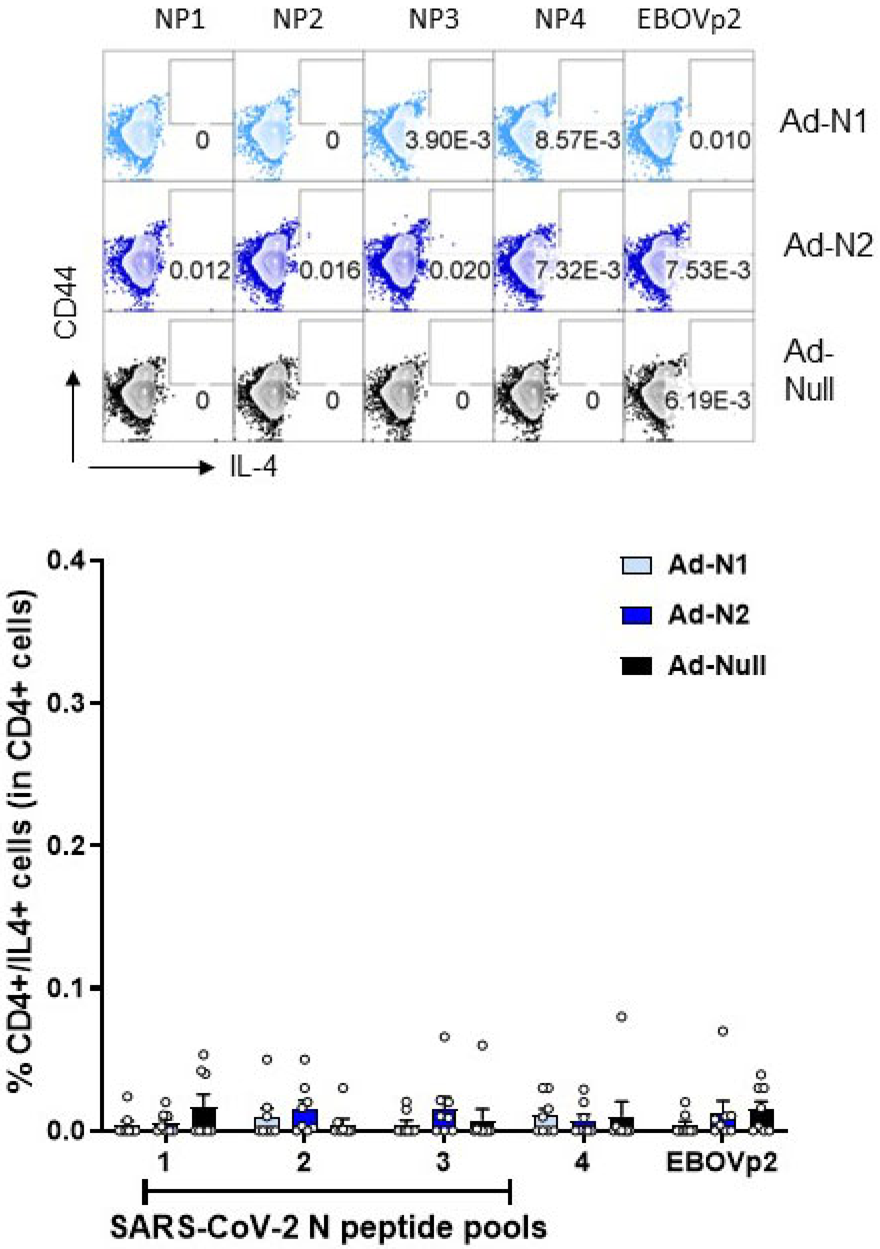

Supplementary Figure 3. Percentage of CD4+/IL4+ cells from splenocytes of immunized mice. Flow cytometry analysis was performed following stimulation with peptides representing the SARS-CoV-2 nucleocapsid protein or the Ebola glycoprotein (EBOVp2). Each dot represents an individual mouse.

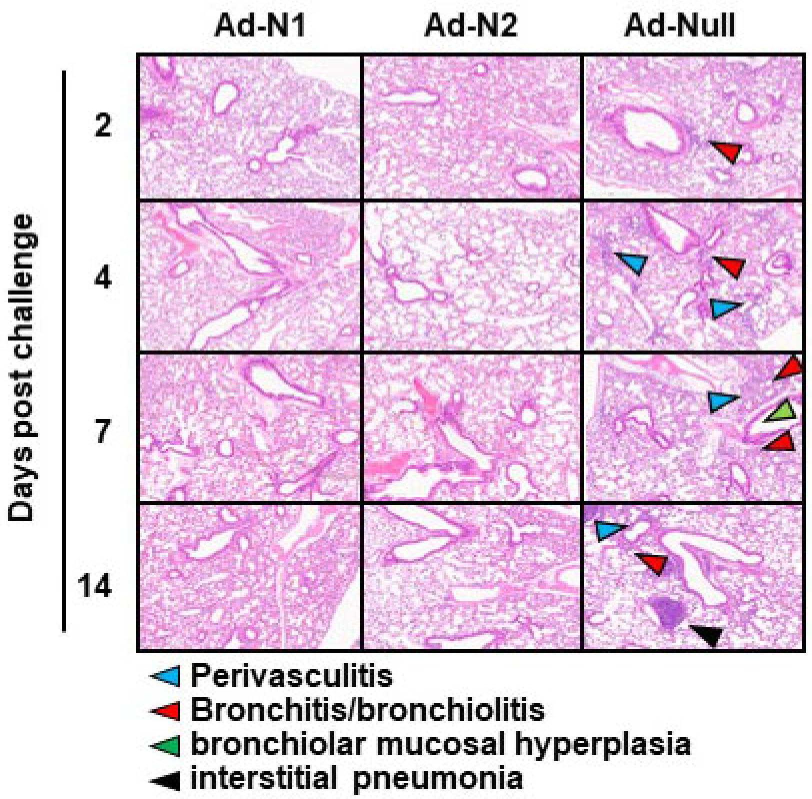

Supplementary Figure 4. Histopathology analysis of Ad-N1, Ad-N2 and Ad-Null immunized mice post-challenge. Selected H&E-stained slides from K18-hACE2 mice post challenge. Arrow heads indicate corresponding pathological findings.

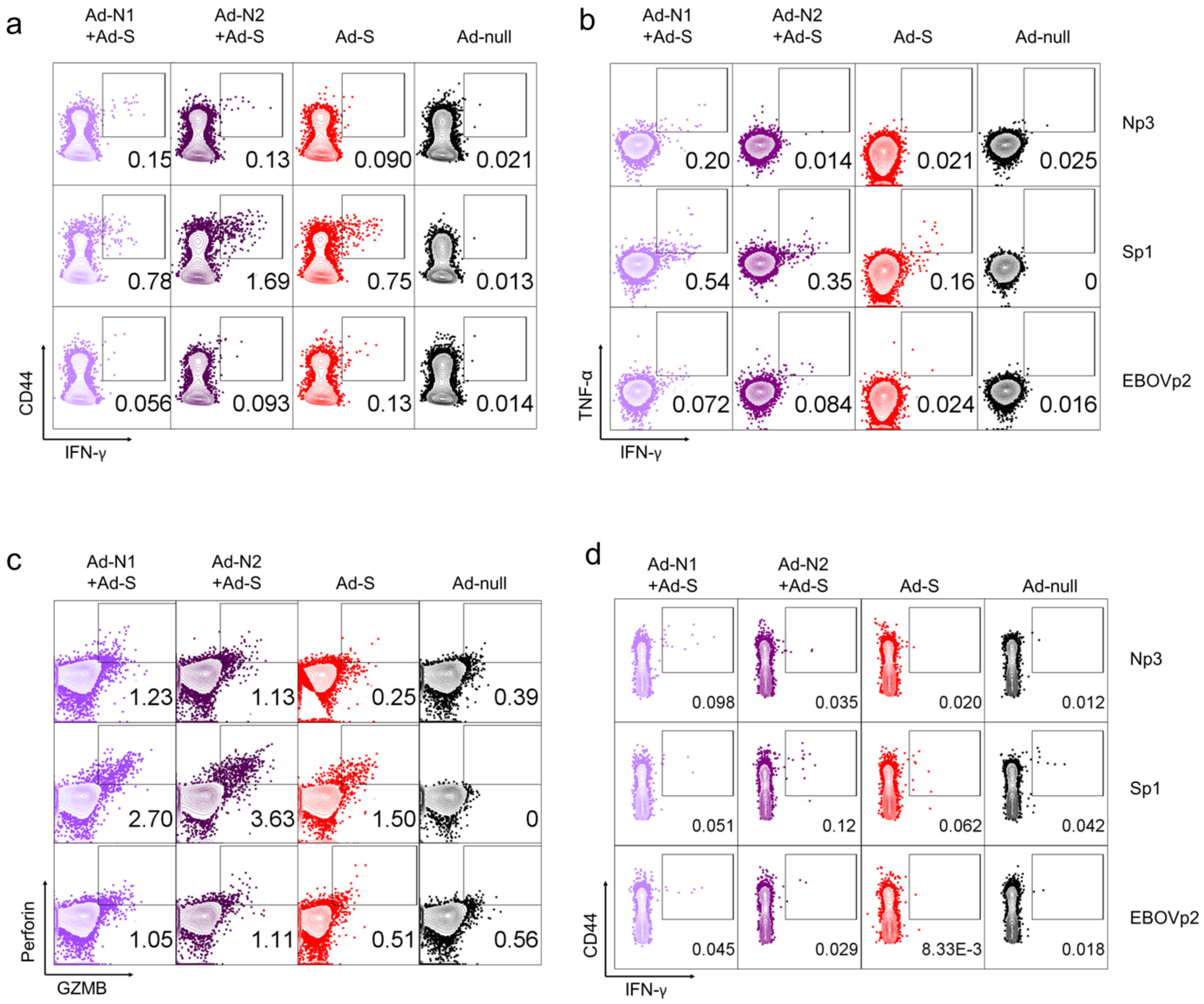

Supplementary Figure 5: Representative flow cytometry dot plots for data shown in Figure 6. Representative flow cytometry dot plots from one mouse per group for data shown in Figures 6a-d. **a**. CD8+ cells expressing IFN-γ following SARS CoV-2 Np3 or Sp1 peptide stimulation. **b**. CD8+ cells expressing both IFN-γ and TNF-α following SARS CoV-2 Np3 or Sp1 peptide stimulation. **c**. CD8+ cells expressing both Granzyme B and Perforin following SARS CoV-2 Np3 or Sp1 peptide stimulation. **d**. CD4+ cells expressing IFN-γ SARS following CoV-2 Np3 or Sp1 peptide stimulation.

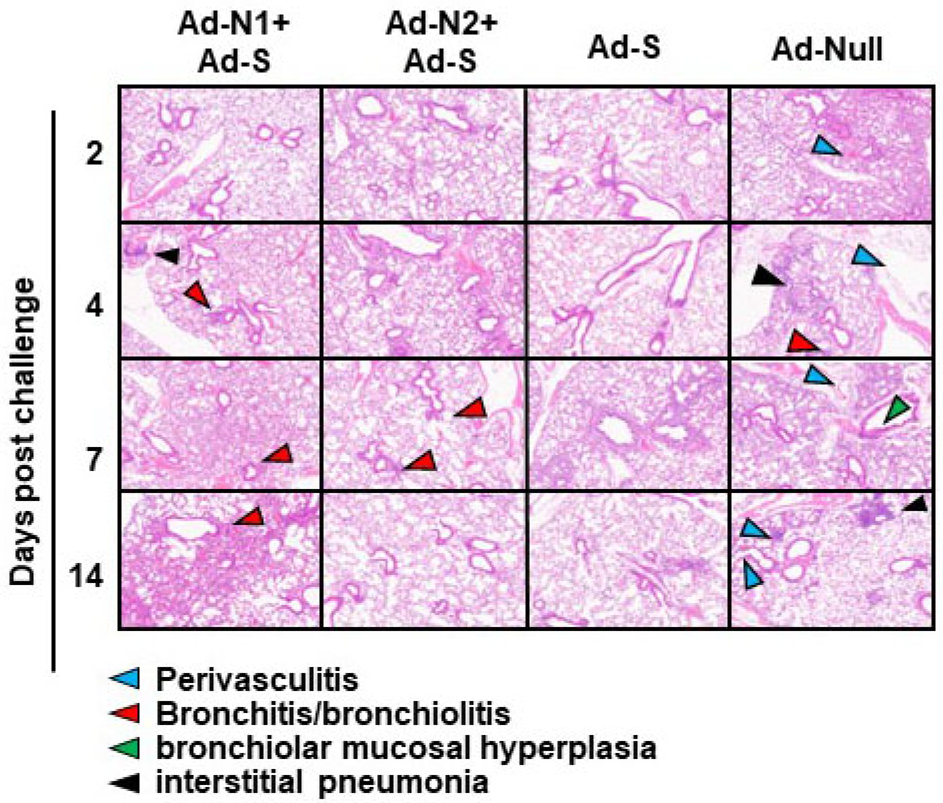

Supplementary Figure 6. Histopathology analysis of Ad-N1+Ad-S, Ad-N2+Ad-S, Ad-S and Ad-Null immunized mice post-challenge. Selected H&E-stained slides from K18-hACE2 mice post challenge. Arrow heads indicate corresponding pathological findings.

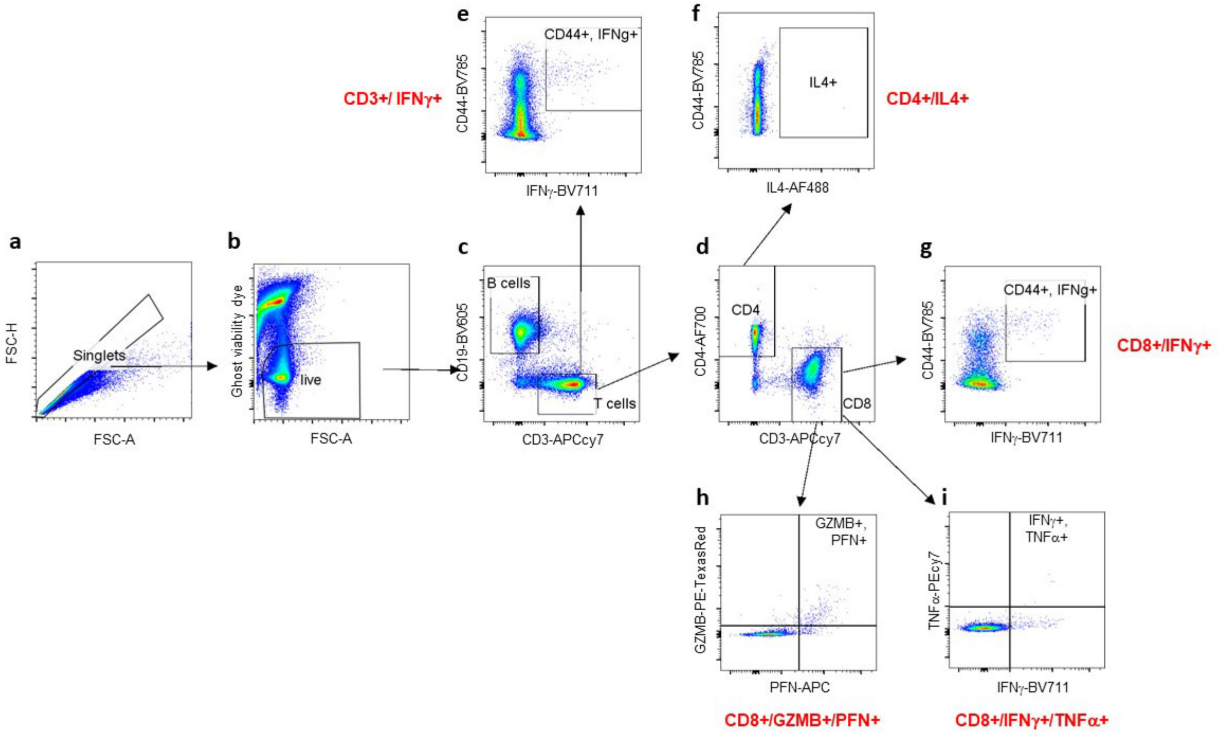

Supplementary Figure 7: Gating strategy for T cell studies. For CD4/CD8 T cell analysis in spleens, the following gating strategy was used. **a**. Singlets were gated with FSC-H and FSC-A. **b**. Live nucleated cells (Live cells) were gated with Live/dead-U450 in singlets. **c**. T cells were gated with CD3-APC-Cy7 and CD19-BV605 in live cells. **d**. CD4 or CD8 T cells were gated with CD4-PE and CD8-BB700 in T cells. **e**. Activated IFN-γ + T cells were gated with CD44-BV785 and IFN-γ-BV711 in T-cells. **f**. Th2 CD4 T-cells were gated with IL4-AF488 in T cells. **g**. Activated IFN-γ + CD8 T cells were gated with CD44-BV785 and IFN-γ BV711 in CD8 T-cells. **h**. Activated cytotoxic CD8 T cells were gated with GZMB-PE-TexasRed and PFN-APC in CD8 T-cells. **i**. Multifunctional CD8 T cells were gated with TNF-α-PEcy7 and IFN-γ-BV711 in CD8 T-cells.
